# Cofilin-1 and Other ADF/Cofilin Superfamily Members in Human Malignant Cells

**DOI:** 10.3390/ijms18010010

**Published:** 2016-12-22

**Authors:** Sergey Shishkin, Lidia Eremina, Natalya Pashintseva, Leonid Kovalev, Marina Kovaleva

**Affiliations:** 1 Laboratory of Biomedical Research, Bach Institute of Biochemistry, Research Center of Biotechnology of the Russian Academy of Sciences, Leninsky Prospekt, 33, bld. 2, 119071 Moscow, Russia; lidia_eryomina@mail.ru (L.E.); pashintseva2009@yandex.ru (N.P.); kovalyov@inbi.ras.ru (L.K.); m1968@mail.ru (M.K.); 2 Department of Biochemistry, Medical Institute, Peoples’ Friendship University of Russia, Miklukho-Maklaya Street, 6, 117198 Moscow, Russia

**Keywords:** cofilin-1, ADF/cofilin superfamily proteins, cell motility, human malignant cells

## Abstract

Identification of actin-depolymerizing factor homology (ADF-H) domains in the structures of several related proteins led first to the formation of the ADF/cofilin family, which then expanded to the ADF/cofilin superfamily. This superfamily includes the well-studied cofilin-1 (Cfl-1) and about a dozen different human proteins that interact directly or indirectly with the actin cytoskeleton, provide its remodeling, and alter cell motility. According to some data, Cfl-1 is contained in various human malignant cells (HMCs) and is involved in the formation of malignant properties, including invasiveness, metastatic potential, and resistance to chemotherapeutic drugs. The presence of other ADF/cofilin superfamily proteins in HMCs and their involvement in the regulation of cell motility were discovered with the use of various OMICS technologies. In our review, we discuss the results of the study of Cfl-1 and other ADF/cofilin superfamily proteins, which may be of interest for solving different problems of molecular oncology, as well as for the prospects of further investigations of these proteins in HMCs.

## 1. Introduction

The key features of malignant neoplasms include uncontrolled proliferation, as well as the ability to invade surrounding tissues (invasion) and to spread locally and regionally or even to distant parts of the body (metastasis). These features are the basis for ideas (which appeared in the 19th century) about the common origin of malignant tumors from stem cells [1,2] and for revealing typical patterns that are associated with tumor phenotypes [3], in particular, by using different OMICS technologies [4]. Nevertheless, malignant tumors vary by tissues of origin and types of differentiation. Moreover, there is a body of evidence that the majority of malignant tumors have intratumoral cell heterogeneity, i.e., are composed of multiple clonal subpopulations of tumor cells with heterogenic morphology that differ on functional properties, in particular on invasive and metastatic potential. Accordingly, malignant tumors can significantly differ by gene expression patterns, including those that are involved in the regulation of proliferation, invasion and metastasis [5,6,7].

The invasion and metastasis are considered to be caused by the dysregulation of motility of malignant cells (see, e.g., Bravo-Cordero et al. [8] and Martin et al. [9]). The accumulated data suggests that changes in cell motility can be triggered by certain actin-binding proteins (ABPs) which provide the formation, function, and restructuring of the actin cytoskeleton [10,11,12,13]. The detection of these changes allows the clarification of the molecular basis of malignant transformation and the role of certain proteins (in particular, ABPs) in this process [11,12,13], and, in addition, allows the list of potential tumor biomarkers to expand [14,15,16].

Over the past ten years coflin-1 (Cfl-1) has attracted special attention among ABPs [14,16,17], along with other members of the actin-depolymerizing factor (ADF)/cofilin superfamily [18,19]. In particular, in our previous studies of the proteomic profiles of different human malignant cells (HMCs), Cfl-1 was almost always found as one of the major proteins [15], and that fact is reflected in the Russian proteomic databases [20,21]. As a consequence, it is interesting to analyze some current trends in the study of ADF/cofilin superfamily members.

## 2. Actin-Depolymerizing Factor/Cofilin Superfamily

### 2.1. From First Actin-Depolymerizing Proteins to Actin-Depolymerizing Factor/Cofilin Superfamily

In the 1980s, several different proteins with actin-depolymerizing activity were identified in vertebrates [22,23]. According to various authors, actin-depolymerizing proteins were characterized by molecular weight (MW) ~19 kDa [22] or ~93 kDa [23]. Almost at the same time proteins with MW ~19 kDa became known as cofilins for their ability to form cofilaments with actin [24]. A similar protein with low MW was termed destrin (destroys F-actin; Dstn), or ADF (e.g., Vartiainen et al. [25] and UniProt P60981). An actin-depolymerizing protein with MW ~93 kDa proved to be gelsolin [23,26]. Confusingly, the alternative name ADF is sometimes used for gelsolin as well as for destrin (UniProt P06396). Three closely related actin-depolymerizing proteins that are usually identified in most vertebrates, Cfl-1, Cfl-2, andDstn (ADF), are often referred to as traditional cofilins. In the late 1990s, traditional cofilins and some related proteins found in different species began to be regarded as a special family, called the ADF/cofilin family [25,27,28].

**Figure 1:**
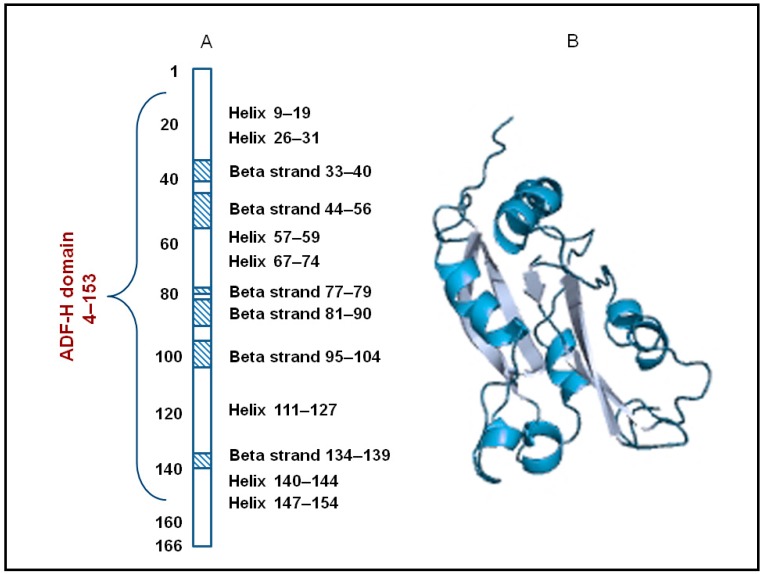
Structure of human cofilin-1 (Cfl-1). (A) Scheme of secondary structural elements identified in the actin-depolymerizing factor homology (ADF-H) domain (amino acid residues 4–153) of human Cfl-1. The dashed boxes show the regions corresponding to β-strands. (B) Ribbon diagram of the human Cfl-1 structure (adapted from PDBsum [30], PDB ID: 1q8x).

At the turn of the 20th–21st centuries Lappalainen et al. found special actin-binding modules of about 150 amino acid residues in polypeptide chains of ADF/cofilins [29]. These modules formed specific three-dimensional structures with six-stranded mixed β-sheets. The abovementioned modules were named actin-depolymerizing factor homology domains, or ADF-H domains (Figure 1).

Lappalainen et al. used the results of genome sequencing of different species that appeared in public databases in order to search for proteins containing ADF-H domains [29]. They identified 39 proteins with sequence similarity to ADF/cofilins. The analysis of primary structures of these proteins showed that they could be subdivided into three structurally distinct classes: ADF/cofilins, twinfilins, and developmentally regulated brain proteins (drebrins)/actin-binding proteins 1 (Abp1s) [29]. In addition to three traditional cofilins, the first class included actin-depolymerizing protein (depactin) from *Asterias amurensis* [31], coactosin from *Dictyostelium discoideum* [32] and actophorin from *Acanthamoeba* [33]. The second class consisted of proteins which were named by Lappalainen et al. as twinfilins due to the fact that their amino acid sequences contained two ADF-H domains [29]. Twinfilin genes were originally published in 1994 and 1997 as genes encoding A6 protein tyrosine kinases. In 1998, the protein was initially identified in *Saccharomyces cerevisiae*, and its sequence homology to ADF/cofilin proteins was demonstrated [29,34]. Later, twinfilin homologues were found in other eukaryotes, except in plants. The third class was composed of drebrins and Abp1s. Drebrins [35] were initially found in chick brain and considered as neuron-specific F-actin-binding proteins able to provide the plasticity of cytoskeleton and to serve as intracellular regulators of morphogenesis [36]. Abp1s are the proteins initially found in yeast. At the beginning of the 21st century the presence of Abp1s in mammals, including humans, was shown [37,38]. Analysis of the sequence alignments of the ADF-H domains allowed the production of the first phylogenetic trees for three classes of ADF-H domain proteins, which indicated the existence of their common ancestral protein and ancestral gene [25,29].

Further investigations resulted in a considerable extension of data on ADF-H domain proteins. In the 2000s, new ADF-H domain protein was detected in the tissues of some vertebrates and became known as glia maturation factor (GMF) [39]. Nakano et al. referred GMF and related proteins to the GMF-family [40]. The first proteins from this family became known in the 1970s–1980s [41,42]. They had capacity to serve as a growth regulator for neurons and glia. In the 1990s with the use of DNA technologies, genes and transcripts encoding proteins closely related to the GMF were discovered in humans. These proteins were called glia maturation factors beta (GMF-B) and γ (GMF-G) (UniProt P60983, O60234).

In 2010, Nakano et al. proposed the consideration of all ADF-H domain proteins as members of a single protein superfamily [40], the ADF/cofilin superfamily. These authors built a new expanded phylogenetic tree for four protein families and one individual group of ADF-H domain proteins. This phylogenetic tree was created based on structural and functional data and, in particular, on the ability to bind monomeric G-actin and polymeric F-actin.

### 2.2. Classification, Structure, and Actin-Binding Properties

Classification of ADF/cofilin superfamily members relies on their structural (amino acid sequences, ADF-H domains) and functional (actin binding) features. According to Nakano et al. [40], there are five groups of ADF-H domain proteins. Two of these groups—twinfilins (bind to G-actin and cap the barbed ends of actin filaments) and drebrin/Abp1s (only bind to F-actin)—fully coincided with two classes described previously by Lappalainen et al. [29]. Unlike Lappalainen et al. [29], Nakano et al. [40] additionally described the group of GMF-family members (GMF-G can bind F-actin) and did not include coactosin and coactosin-like proteins, which only bind to F-actin, in the group of ADF/cofilins (that bind to both F- and G-actin and promote actin depolymerization).

**Figure 2:**
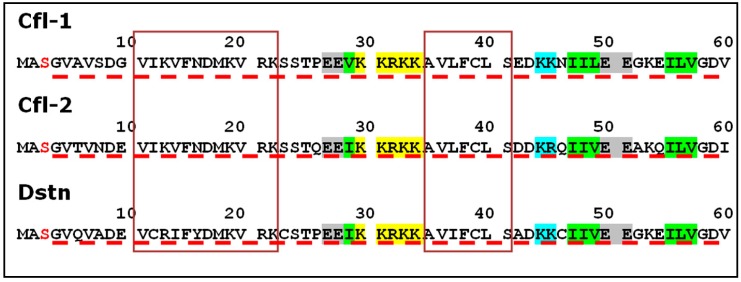
N-termini of human traditional cofilins Cfl-1, Cfl-2 and destrin (Dstn) according to UniProt (P23528, Q9Y281, P60981, respectively). Red “S” indicate serine residues which can be phosphorylated. Identical regions of amino acid sequences are framed. Nuclear localization signals are labeled in yellow. Repeating hydrophobic amino acid residues are labeled in green. Repeating positively charged amino acid residues are labeled in blue. Repeating negatively charged amino acid residues are labeled in gray. Starting parts of ADF-H domains (see below) are shown by the dotted red lines.

The first group (ADF/cofilins) consists of proteins that were identified in different organisms ranging from *S. cerevisiae* to *Homo sapiens*. It includes traditional cofilins (Cfl-1, Cfl-2, Dstn), depactin, and actophorin. These proteins with a MW of about 20 kDa have a significant structural similarity. Each of them consists of ADF-H domain with a few additional amino acid residues (including initiatory methionine) at the N-terminus of the polypeptide chain and about ten amino acid residues at the C-terminus (according to UniProt; Table 1). The proteins referred to as traditional cofilins have been detected in a variety of vertebrates and are the most studied members of the ADF/cofilin superfamily. For human cofilins the tissue specificity has been demonstrated. Cfl-1 encoded by *CFL1* gene is widely distributed in various tissues and is named non-muscle isoform (UniProt P23528). Cfl-2—muscle isoform—may exist in at least two variants due to alternative splicing of a single gene *CFL2* [43]. One of these isoforms (Cfl-2b) is present in skeletal muscle and heart, and the other (Cfl-2a) has been revealed in various tissues (see also UniProt Q9Y281). Dstn encoded by *DSTN* gene is also widely distributed in various tissues (UniProt P60981). ADF/cofilins can bind F-actin and sever actin filaments. On the one hand, severing of the actin filament causes actin depolymerization. On the other hand, it can lead to actin polymerization directly or indirectly by producing free barbed ends [44]. Along with binding of F-actin, ADF/cofilins have the ability to bind G-actin in a 1:1 ratio [24,29]. It is currently believed that the molecules of the traditional ADF/cofilins have two distinct actin-binding sites, the G/F-site located in the C-terminus and the F-site located in the N-terminus. The F-site is involved in the binding of F-actin, and the G/F-site is required for binding to both the G-actin and the F-actin [45]. The functionally important amino acid residues at the N-terminal end of the human cofilins are shown in Figure 2. ADF/cofilins bind preferably to ADP-forms of G- or F-actin and use energy from ATP hydrolysis in actin polymerization [46]. It has been demonstrated that cofilin can directly bind not only to actin, but also to phosphatidylinositol 4,5-bisphosphate (PIP2) [47] and to serine/threonine-protein kinase LIMK1 [48]. ADF/cofilins from vertebrates are found to contain nuclear localization sequences (see Figure 2 and UniProt P23528).

The proteins from the second group (twinfilins) have two tandem ADF-H domains that are located near the N-terminus of the polypeptide chain and are separated by a linker area of several dozen amino acid residues. Typical twinfilins have a MW of about 40 kDa. It has been shown that at least in humans, mice, and *S. cerevisiae,* twinfilins are presented by two isoforms, each of which are encoded by their own gene (e.g., *TWF1* and *TWF2* in human, according to UniProt Q12792 and Q6IBS0). Additionally, in mice, an alternative promoter is responsible for production of two proteins: TWF-2b in striped muscles (heart and skeletal muscles) and TWF-2a mainly in non-muscle tissues and organs [49]. Twinfilins can interact with G-actin forming 1:1 complexes, and some of the twinfilins can bind F-actin, as well. In mammals, two ADF-H domains of twinfilins allow both capping of the barbed end of actin filaments and sequestering of actin monomers [50].

The third group is composed of drebrins and Abp1s, proteins with a single ADF-H domain, but with higher MW (~70 kDa) than the traditional cofilins and twinfilins. Drebrins are typical for vertebrates. Three isoforms—embryonic (E1 and E2), and adult (A)—have been found to be generated by alternative splicing from a single gene *DBN1*. In humans, drebrins are presented in brain neurons and also in the heart, placenta, skeletal muscle, kidney, pancreas, peripheral blood lymphocytes including T-cells (see [51] and UniProt Q16643). Abp1 proteins have a slightly lower MW than drebrins, but a similar primary structure. Abp1s have been found in mammals, including humans [37,38]. The human Abp1 protein has a MW of 48 kDa and the structure of the polypeptide chain which is very similar to the structure of typical drebrins. This fact has served as the basis for the recommended name of this protein— drebrin-like protein (synonyms hematopoietic progenitor kinase 1-interacting protein of 55 kDa (HIP-55), drebrin-F) (UniProt Q9UJU6). Drebrins and Abp1s have a single ADF-H domain in their N-termini, followed by a nonconserved central region and a C-terminal region. These proteins have been shown to bind F-actin and stabilize actin filaments. Some proteins of this group (but not human drebrin) have a C-terminal Src homology 3 (SH3) domain [50]. 

The fourth group is presented by the GMF-family proteins. These proteins have a small MW (14–17 kDa). GMF has been found in the tissues of some vertebrates. Despite the presence of ADF-H domain, GMF is not able to directly bind actin. GMF-B that is present in the brain of all vertebrates is also not able to bind actin (UniProt P60983). GMF-G is present predominantly in lung, heart, and placenta (e.g., [52] and UniProt O60234). It has structural similarity to GMF-B; however, unlike GMF-B, it was found to interact with F-actin [52,53]. Goroncy et al. analyzed the structure of ADF-H domains of GMF proteins. The authors obtained recombinant mouse GMF-B and GMF-G proteins, and studied their structures using nuclear magnetic resonance spectroscopy [39]. Both GMF structures displayed two additional β-strands in one of the loops. These β-strands were not seen in the protein structures of other ADF-H classes, thus, according to Goroncy et al. [39], these β-strands may be a class-defining feature. Both GMF-B and -G can interact with the actin-related protein 2/3 (Arp2/3) complex, inhibit its activity and induce actin disassembly [40]. Another member of GMF-family, GMF1, the yeast protein discovered by Nakano et al. [40], is also able to interact with the Arp2/3 protein complex and to suppress its activity.

The fifth, separate, group includes coactosin from *D. discoideum* and coactosin-like proteins (from different species including that of *H. sapiens*—UniProt Q14019). These proteins are entirely composed of a single ADF-H domain and have a MW (about 17 kDa) similar to the MW of traditional cofilins. However, unlike ADF/cofilins, coactosin and coactosin-like proteins bind only F-actin and do not promote actin depolymerization [40,50]. Moreover, some antagonistic relations between the traditional cofilins and coactosin-like 1 protein have been reported [54]. Interestingly, coactosin from *Entamoeba histolytica* has been recently described as an unusual type of coactosin which binds both F- and G-actins [55].

Some characteristics of the main human ADF/cofilin superfamily members are summarized in Table 1.

### 2.3. Biological Functions

Traditional cofilins, the most well-studied members of the ADF/cofilin superfamily, are known to modulate actin dynamics by catalyzing actin depolymerization or polymerization through the severing of actin filaments. The effect of cofilins on actin filaments (assembly or disassembly) depends on the concentration of active cofilins, the relative concentration of G-actin, and some protein factors. In low concentrations, ADF/cofilins sever the actin filaments and promote depolymerization. High concentration of cofilins is suggested to promote actin nucleation and polymerization [64]. Cofilins can contribute to actin polymerization producing free barbed ends and supplying actin monomers. Cfl-1 is currently understood to modulate actin nucleation and filament branching through synergy or competition with the Arp2/3 complex. The Arp2/3 protein complex is a seven-subunit complex of actin-related proteins that enables binding to actin, providing nucleation and formation of actin branches [65,66]. The formation of actin branches is one of the key events of the production of lamellipodia, which are essential for cell motility. Cfl-1 and Arp2/3 have been shown to work in synergy (i.e., with a cooperative effect) producing free barbed ends for actin polymerization [67]. In parallel, Cfl-1 can reduce the affinity of the Arp2/3 complex for filaments and promote dissociation of old actin branches [68]. Cfl-1 and Cfl-2 have also been shown to regulate the assembly of actomyosin complex blocking the binding of tropomyosin and myosin II to actin filaments [24,69]. It was found that, in vivo, cofilins participated in the reorganization of actin cytoskeleton in response to stresses and different cell stimuli [70]. Overexpression of cofilins leads to the formation of stress fibers, contractile actin bundles that have been found in non-muscle cells and shown to play an important role in cellular contractility, providing cell adhesion, migration (including assembly of lamellipodia and filopodia), and morphogenesis [71]. Due to this function, cofilins are regarded as molecular regulators of development processes. Cfl-1 and destrin are required for ureteric bud branching morphogenesis [72]. According to Sparrow et al. [73], Cfl-1 is necessary for dynamic changes in the cytoskeleton needed for axon engagement and is essential for Schwann cell myelination. Evidence for the involvement of Cfl-1 (and the Arp2/3-complex) in the regulation of axonal growth cones has been recently reviewed by Dumpich et al. [74]. Cofilin can also participate in regulation of cell proliferation in response to mechanical stresses. In mammalian epithelial cells it inhibits through the cytoskeleton remodeling activity of Yes-associated protein 1 (YAP1) and Translin-associated zinc finger protein 1 (TAZ1), mediators of Hippo signaling pathway and organ growth, thus inhibiting cell proliferation [75]. Numerous data on the participation of Cfl-1 in development are summarized in review [76]. To sum up, ADF/cofilins play an essential role in the controlling of actin dynamics. They have a dual effect on actin filaments and may contribute to cellular contractility through both the local actin depolymerization and the formation of stress fibers, and therefore they are important for morphogenesis and development.

In addition, cofilins have also functions in cells that are not directly related to the regulation of actin dynamics. The first is that cofilins can provide transport of actin molecules (which do not contain the nuclear localization signals) to the nucleus [77]. Using immunofluorescence microscopy, Ono et al. revealed ADF (Dstn) and cofilin in nuclei of cultured myogenic cells and demonstrated the colocalization of ADF and cofilin in intranuclear actin rods [78]. G-actin which is transported to the nucleus by means of Cfl-1 may act as a key player for nuclear structure and function regulating both chromosome organization and gene activity (e.g., see [79]). Сofilin has been characterized as a connecting link between T-cell co-stimulation and actin translocation to the nucleus [80,81]. Co-stimulatory signals from ligand attachment to accessory receptors like the cluster of differentiation 2 (CD-2) are required for the production of the T-cell growth factor interleukin 2 (IL-2) and cell proliferation. In T lymphocytes, cofilin is a component of the costimulatory signaling pathways: CD-2 stimulation leads to dephosphorylation of cofilin, binding to G-actin and translocation into the nucleus [80,82]. In addition to G-actin, Cfl-1 is also able to transport to the nucleus various regulatory proteins that affect the processes of transcription (e.g., Runt-related transcription factor 2 (Runx2)) and cell differentiation [83]. The other function of cofilins that is not related to the regulation of actin dynamics is their participating in apoptosis. Cofilin oxidation and translocation to the mitochondrion has been found to induce apoptosis through the opening of the mitochondrial permeability transition pore and release of cytochrome c [84]. At last, Cfl-1 has been shown to directly activate phospholipase D1 which is important for cell migration [85,86].

The essential roles of traditional cofilins (Cfl-1, Cfl-2 and Dstn) in mammals have been proved by experiments on cofilin/ADF-knockout mouse strains [87,88,89]. In such experiments, the homozygous mice *Cfl-1*^−/−^ were embryonic lethal while heterozygous mice *Cfl-1*^+/−^ were viable. It was shown that Cfl-1 was not essential for the extensive morphogenetic movements during gastrulation, because the other proteins (e.g., Dstn) can provide cellular contractility instead of Cfl-1 at this stage of embryogenesis. However, the *Cfl-1* knockout at later stages dramatically altered the processes of neuronal development. Although Dstn was overexpressed in mutant embryos *Cfl-1*^−/−^, this could not compensate for the lack of Cfl-1, suggesting that these proteins might have a different function in embryonic development. Mice lacking ADF were viable and had no alterations during embryonic development [87]. The *Cfl-2* knockout led to severe protein aggregate myopathy in a mouse model [89].

The various cellular functions of traditional cofilins including those in regulation of nuclear integrity and transcriptional activity, apoptosis, nuclear actin monomer transfer, and lipid metabolism are discussed in recent review of Kanellos and Frame [90].

**Figure 3:**
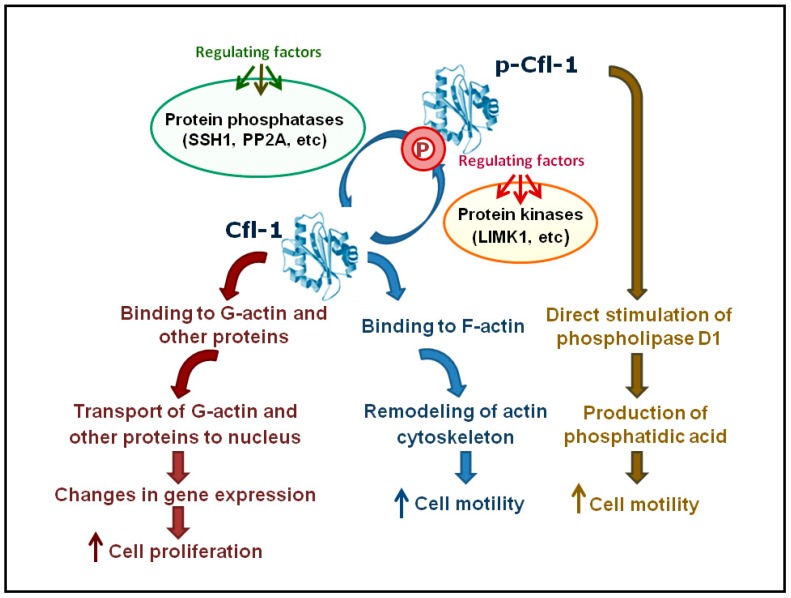
Schematic model summarizing Cfl-1 functions in vertebrates. Regulating factors (green) and regulating factors (red) refer to different signaling pathways, leading to Cfl-1 dephosphorylation (activation) or phosphorylation (inactivation), respectively. p-Cfl-1: phospho-Cfl-1; SSH1: Slingshot protein phosphatase 1; PP2A: Protein phosphatase 2A; LIMK1: LIM domain kinase 1.

A schematic model summarizing the Cfl-1 functions in vertebrates is shown in Figure 3.

Similarly to cofilins, twinfilins are also involved in the regulation of actin dynamics and can participate in formation of cellular protrusions such as lamellipodia and filopodia in collaboration with other actin binding proteins (Arp2/3, cortactin, etc.) [91]. In *Drosophila* twinfilin is required for cell migration and endocytosis. In mammalian cells, TWF-1 is also involved in endocytosis and migration, and participates in cell morphogenesis [50]. TWF-2a is shown to be involved in the morphogenesis of neurons. *TWF-2a* knockout mice developed normally without any abnormalities, due to the fact that it is typically co-expressed in the same tissues with TWF-1 and has similar function [92]. The specific role of TWF-2b, which is expressed exclusively in heart and skeletal muscles, is currently unclear.

Drebrin and Abp1 have been shown to regulate actin filament organization, especially during development of neuronal cells. Drebrin E is highly abundant in the developing brain. This protein may modulate actomyosin interaction within dendritic spines and alter spine shape [93]. Similarly, drebrin (isoform E) is involved in the regulation of axonal growth through actin-myosin interactions [94]. Drebrin E regulates neuroblast migration in the postnatal mammalian brain [95]. Drebrin A predominates in neurons of the adult forebrain. Neuronal drebrin (isoform A) inhibits cofilin-induced severing of F-actin due to direct competition between these two proteins for F-actin binding [96]. Drebrin (E2 isoform) has been also found in various non-neuronal cells, including fibroblasts, stomach and kidney epithelia [97], and keratinocytes [98], where it plays a role in, for example, adhering junctions. Abp1 is shown to be implicated in endocytotic processes. It uses C-terminal SH3 domain to bind various proteins including regulators of endocytosis. Particularly, it associates with dynamin, a large GTPase essential for vesicle fission [99]. Due to its ability to interconnect the actin cytoskeleton and participate in endocytosis, Abp1 regulates lymphocyte and leukocyte responses [38,50,100].

GMF does not bind actin, but binds Arp2/3 complex and suppress its activity which results in stimulation of filament debranching and inhibition of actin nucleation [101]. Nakano et al. described the blocking of the Arp2/3 complex by GMF1 protein as a reason for the modulatory effect of GMF1 on the yeast actin cytoskeleton [40]. GMF has been shown to regulate lamellipodial protrusion dynamics and cell migration [102]. GMF-B including the human one is also not able to bind actin. To date, it has been established that GMF-B induces synthesis of some proinflammatory cytokines, as well as influences the differentiation and aging of various cells of the nervous system in normal and pathological conditions (e.g., see [103,104] and UniProt P60983). In fibroblasts, GMF-B controls branched actin content and lamellipodial dynamics [105]. The main function of GMF-G is still unclear. This protein found predominantly in lung, heart, and placenta is capable of interacting with F-actin and influencing cell motility [52,53]. 

Functions of coactosin and coactosin-like proteins are insufficiently understood. It has been shown that coactosin inhibits barbed end capping of actin filament and is involved in actin polymerization. The knockdown of coactosin has resulted in the disruption of actin polymerization and of neural crest cell migration [106]. In chick embryos, coactosin was expressed during morphogenetic movement and associated with actin stress fibers in cultured neural crest cells [107]. In vitro studies demonstrated that coactosin-like protein can protect F-actin from cofilin-mediated depolymerization [54]. Additionally, coactosin-like protein is known to support the activity of 5-lipoxygenase, an enzyme involved in leukotriene biosynthesis. Coactosin-like protein binds 5-lipoxygenase and translocates it from cytosol to the nucleus. In coactosin-like protein knockdown human monocytic cell line, the activity of 5-lipoxygenase is decreased, but not absent [108].

### 2.4. Regulaton

The activity of ADF/cofilin superfamily members is regulated by various mechanisms. ADF/cofilins are shown to be regulated by pH, phosphatidylinositols, protein kinases, and phosphatases, as well as some other proteins. Moreover, their activities can depend on cellular redox status.

It is well known that F-actin binding and depolymerizing activity of cofilins depends on pH. Yonezawa et al. reported that in vitro, in an F-actin containing model system, at pH < 7.3 the concentration of monomeric actin (G-actin) was less than 1 µM, even with an excess of cofilin added [109]. However, at pH > 7.3 the concentration of G-actin increased proportionally to the concentration of cofilin added, until the complete depolymerization of F-actin. The authors formed the conclusion that cofilin is capable of reversibly controlling actin polymerization and depolymerization in a pH-sensitive manner. Later, pH was demonstrated to modulate cofilin activity in vivo [110]. However, pH sensitivity is apparently not a common feature of all ADF/cofilins in all species. For example, mouse Cfl-1 unlike human has been shown to be pH-independent, as well as mouse Cfl-2 [25].

Membrane phosphoinositides, particularly PIP2, are also known to regulate ADF/cofilin activity. Cofilins can directly bind phosphatidilinositols, and PIP2-binding area on the surface of the cofilin molecule overlaps with the actin-binding site [47]. Therefore, binding to PIP2 leads to inhibition of ability to bind to actin. Changes in PIP2 density of the cellular membrane can regulate a balance between membrane-bound and free active ADF/cofilins [111]. 

Phosphorylation of Cfl-1 on a serine residue (Ser3) inhibits its binding to F- and G-actin [112]. Similar data were obtained for Dstn [113]. Only dephosphorylated (active) cofilin can carry out the functions associated with binding of actin and protein translocations to the nucleus and mitochondrion. In contrast, phosphorylated cofilin is required to activate phospholipase D1 [114]. The regulation of cofilins by phosphorylation/dephosphorylation is performed via signaling pathways involving kinases and phosphatases in response to extracellular signals and changes in microenvironment [14,17,115]. In mammals, Cfl-1 has been shown to be phosphorylated and inactivated by LIM-kinases (LIMK1, LIMK2) and testicular protein kinases (TESK1, TESK2). Conversely, cofilin is dephosphorylated and activated by slingshot protein phosphatases (SSH1, SSH2, SSH3), protein phosphatases 1 and 2A (PP1, PP2A), and chronophin (CIN) (for a review, see [75]). Reactions of the phosphorylation/dephosphorylation of cofilins have a significant impact on modulation of actin dynamics, thus influencing cell motility and morphogenesis in vertebrates [116,117]. For this reason, kinases and phosphatases of cofilins may play a crucial role in the development. The overexpression of LIMK1 or inactivation of SSH1 results in abnormal accumulation of F-actin and incorrect cytogenesis during mitosis [118]. Since LIMK1 inactivates cofilin, it has been thought to downregulate lamellipodium formation and inhibit cell migration [119]. However, treatment of Jurkat T cells with LIMK1 inhibitor has been shown to block stromal cell-derived factor (SDF) 1α-induced chemotaxis of T cells [120]. It has been assumed that LIMK1-catalyzed phosphorylation of cofilin is essential for chemotactic response of T lymphocytes, but the results from Condeelis’ group, who showed that non-phosphorylatable mutant cofilin provides the generation of protrusions and determines the direction of cell migration, have contradicted the fact that phosphorylation and inactivation of cofilin are crucial for cell motility [121]. Nevertheless, further experiments confirmed the positive role of LIMK1 in migration of chemokine-stimulated Jurkat T cells. The cell migration turned out to be suppressed by LIMK1 knockdown, whereas knockdown of SSH1 causes the formation of lamellipodia around the periphery of the cell after cell stimulation [122]. Thus, it has been proposed that LIMK is required for generation of multiple lamellipodia in the initial stages of the cell response, and SSH1 is needed to restrict lamellipodial protrusions for directional cell migration [123]. In fact, although LIMK seems to be a positive regulator of cell migration, mechanisms for this regulation are still not completely understood.

Apart from the kinases and phosphatases already described, the interaction of ADF/cofilins with actin can be directly or indirectly regulated by a wide range of other proteins. The binding of cofilin to cortactin is one of the mechanisms of cofilin inactivation which is typical for podosomes and invadopodia, actin-based dynamic protrusions produced by invasive cancer cells, vascular cells, and macrophages [124,125]. Actin-interacting protein 1 (AIP1) and cyclase-associated protein 1 (CAP1) promote the disassembly of cofilin-bound actin filaments [126,127]. Coronin provides recruiting cofilin to filament sides and thus enhances actin filament severing [128]. The Rho GTPases are important regulators of actin dynamics, including stress fiber formation, and are involved in the regulation of ADF/cofilins via LIMK. RhoA activates Rho-associated coiled-coil forming kinase (ROCK) which can phosphorylate and activate LIMK. Thus, RhoA stabilizes the stress fibers and prevents depolymerization of actin filaments through the phosphorylation of cofilin, and Rho–ROCK–LIMK–cofilin pathway modulates actin assembly in various cell types in response to extracellular stimuli [76]. Epidermal growth factor (EGF) has been shown to influence cofilin through the LIMK pathway or phospholipase C-mediated hydrolysis of PIP2 and release of cofilin from membrane sequestering [129]. The mechanisms which include activation of cofilin and generation of free barbed ends for lamellipodial extension in response to EGF stimulation have been described mainly for migrating malignant cells [129,130]. However, the increase of cofilin-dependent severing activity after stimulation with EGF does not always correlate with the level of dephosphorylated cofilin [131], indicating a more complex regulatory mechanism than previously thought.

The cellular redox state may play an important role in regulating ADF/cofilins. This regulation is performed by oxidative post-translational modifications of Cys residues including *S*-glutathionylation [132], disulfide bonds [133], and *S*-nitrosylation [134]. Redox-related modifications influence cofilin activity and signaling pathways with its participation. Cofilin is found to be a target of oxidation under oxidative stress in T cells. Cofilin oxidation leads to formation of intramolecular disulfide bonds and to dephosphorylation at Ser3. Although dephosphorylated oxidized cofilin is still able to bind to F-actin, it cannot perform actin depolymerizing function, and the F-actin level increases [133]. Instead, oxidized cofilin acquires the ability to translocate actin to the mitochondria, where it induces cytochrome c release by opening of the permeability transition pore. As a result, mitochondrial damage and apoptosis are induced [84].

Thus, the cellular microenvironment (namely pH, phosphoinositides and proteins including enzymes) can essentially influence cofilin functions. The other members of the ADF/cofilin superfamily have been shown to share some of these aspects of regulation. However, there are few available data addressing possible mechanisms of their regulation. Twinfilins have been demonstrated to promote filament severing in a pH-dependent manner. As opposed to ADF/cofilins, TWF-1 severs actin filaments in vitro at pH below 6.0 [135]. Twinfilins can bind PIP2 similarly to ADF/cofilins, and this interaction down-regulates the actin binding, filament severing, and actin monomer sequestering activities [91,92,135]. TWF-1 and TWF-2 bind to capping protein (CP), which has been shown to inhibit directly the severing activity of TWF-1 [135]. The small GTPases Ras-related C3 botulinum toxin substrate 1 (Rac1) and cell division control protein 42 homolog (Cdc42) induce the localization of TWF-1 to membrane ruffles and cell-cell contacts, but do not affect the localization of TWF-2 [91]. Drebrin phosphorylation by cyclin-dependent kinase 5 (Cdk5) regulates cytoskeletal reorganization associated with neuronal migration. Drebrin E can be phosphorylated on Ser142, and drebrin A on Ser142 or Ser342 [136]. Localization of drebrin to the distal part of axonal filopodia and branching in drebrin overexpressing neurons are negatively regulated by myosin II [137]. Likewise ADF/cofilins, GMF-family proteins have been shown to be regulated by phosphorylation. GMF-G phosphorylation at Tyr104 by Abelson tyrosine-protein kinase 1 leads to the dissociation of GMF-G from Arp2/3, reduction of actin disassembly and facilitation of smooth muscle contraction [138]. The subfamily of the Rho GTPases, Rac, is involved in regulation of coactosin activity. In response to Rac signaling, coactosin is recruited to lamellipodia and filopodia, promoting actin polymerization and neural crest cell migration [106].

As a whole, ADF/cofilin superfamily proteins play a multifaceted role in cells. Since they are involved in proliferation and migration of mammalian cells, they can also be implicated in various pathological processes, including tumor growth, invasion, and metastasis. The study of the possible contribution of these proteins to malignant phenotype of cancer cells is an important task of molecular oncology.

## 3. Members of the Actin-Depolymerizing Factor/Cofilin Superfamily in Human Malignant Cells

### 3.1. ADF/Cofilins

**Figure 4:**
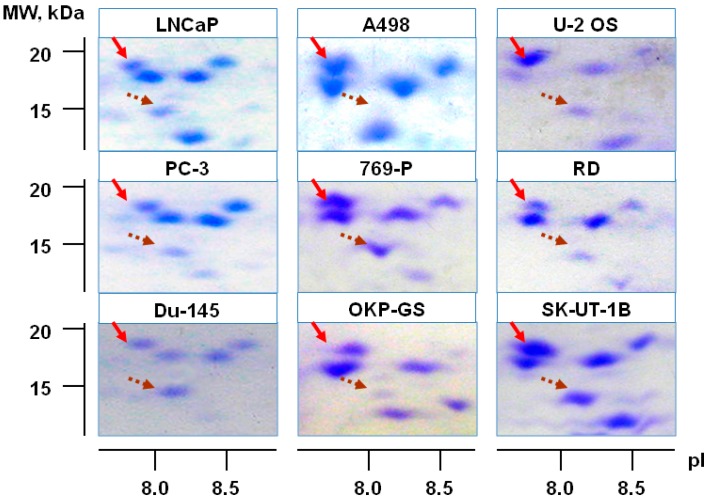
Fragments of two-dimensional electrophoregramms of human malignant cells (HMCs) derived from prostate adenocarcinoma (LNCaP, PC-3, Du-145), renal adenocarcinoma (A498, 769-P, OKP-GS) and sarcoma (U-2 OS—osteosarcoma; RD—rhabdomyosarcoma; SK-UT-1B—leiomyosarcoma) cells lines. The red arrow shows the identified Cfl-1 fraction, and the brown dotted arrow shows the profilin fraction as a reference spot.

To our knowledge, the first report on detection of Cfl-1 protein in HMCs was published by Stierum et al. [139]. Using proteomic technologies (two-dimensional electrophoresis (2-DE) and mass-spectrometric identification) the authors revealed that Cfl-1 was involved in processes of cell differentiation in colorectal adenocarcinoma (Caco-2) cell line. Later, Cfl-1 was identified in different tumor cell lines and tissues including adenocarcinomas [15,140,141,142,143], osteosarcoma [144], lymphoid tissue neoplasms [145], astrocytoma [146], glioma [147], and neuroblastoma [148]. Accordingly, it is possible to think that Cfl-1 is a common participant in various tumor phenotypes. In particular, the results of identification of Cfl-1 in various HMCs are presented in the multi-level information database “Proteomics of malignant cells” [21]. These results for Cfl-1 in several carcinomas and sarcomas cell lines are shown in Figure 4. Cfl-1 is present on 2-DE gels in high quantity (since it is detected by routine Comassie R-250 staining) and can be attributed to 200 of the most abundant proteins of HMCs.

The increased mRNA and protein levels of Cfl-1 in comparison with control (nonmalignant) cells have been shown in various HMCs including those from breast [140], lung [142], prostate [149] etc. Overexpression of Cfl-1 has been mainly associated with tumor cell proliferation, invasion, and metastasis [14,140,150,151]. It has also been suggested that dephosphorylated, active cofilin is increased in HMCs [77,151]. However, there are a few opposing reports. For example, the overexpression of Cfl-1 suppressed growth and invasion of non-small cell lung cancer [152], and the phosphorylation of cofilin was elevated in bladder cancer samples compared with the normal bladder tissues [153]. Many authors have considered Cfl-1 protein as a diagnostic/prognostic tumor biomarker [145,154,155]. Zheng et al. found reliable increasing of Cfl-1 in blood samples obtained from patients with lung adenocarcinoma compared to healthy control [156]. Cfl-1 can be a target for chemotherapeutic treatment. It has been shown that docetaxel induces the apoptosis of prostate cancer cells via suppression of the cofilin signaling pathways [157]. The increased level of Cfl-1 in HMCs is often associated with poor prognosis which can be related with cofilin-dependent drug resistance of cancer cells [142,150,158]. Cfl-1 has been upregulated in multidrug resistant malignant cells compared with non-drug resistant malignant cells [142]. High Cfl-1 levels have been correlated with cisplatin resistance in lung adenocarcinomas [158]. Cfl-1 may serve as a predictor of poor response to platinum-based chemotherapy in human ovarian cancer cells [143] and in astrocytomas cells [146].

The molecular mechanisms of Cfl-1 involvement in the formation of malignant phenotype of cancer cells are still being investigated. In tumor cells, the actin dynamics and cell motility are initiated in response to stimuli in the microenvironment. EGF, as well as transforming growth factor-α (TGFα), stromal cell-derived factor 1 (SDF1) and heregulin have been demonstrated to be involved in stimulation of cell migration and correlated with progression of various tumors [14]. Dephosphorylation and activation of Cfl-1 upon EGF stimulation increases F-actin-severing activity of cofilin and generation of free barbed ends that are required for lamellipodial extension and chemotaxis to EGF, leading to invasion and metastasis [129,159]. Thus, excess of dephosphorylated Cfl-1 may be implicated in malignant phenotype of cells. This concept has been supported by a number of authors [151,160,161]. Particularly, Nagai et al. showed that overexpression of non-phosphorylatable cofilin mutant (cofilin-S3A) in astrocytoma cells resulted in more highly invasive phenotype than those xenographs expressing wild-type cofilin [151]. 

Nuclear translocation of dephosphorylated Cfl-1 can also contribute to malignant phenotype of cells. Dephosphorylated Cfl-1 provides transport of G-actin to the nucleus. Nuclear actin can be involved in chromatin remodeling, transcription, RNA processing, intranuclear transport, nuclear export, and maintenance of the nuclear architecture [162]. Correspondingly, the gene expression changes during cancer progression can be mediated by Cfl-1 through actin transport. Another mechanism contributing to malignant phenotype of cells and related with Cfl-1 dephosphorylation and nuclear translocation was described by Samstag and colleagues [80,82]. In untransformed T lymphocytes, cofilin is part of a costimulatory pathway that is important for the induction of T-cell proliferation (i.e., for production of IL-2). In response to ligand attachment to accessory receptors like CD-2, cofilin undergoes dephosphorylation and nuclear translocation. In malignant T lymphoma cells, dephosphorylation and nuclear translocation of cofilin occur spontaneously through constitutive activation of serine protein phosphatase. These events lead to T-cell proliferation and inhibition of apoptosis [80,82]. 

Cofilin activation/inactivation are modulated by changes in balance of kinases, phosphatases and other cofilin upstream regulatory proteins. These changes are responsible for initiation of the early steps of cancer cell motility and metastasis [119]. SSH1 is the most well-studied cofilin phosphatase which has been found to be upregulated in various invasive cancer cells. Wang et al. have revealed that overexpression of slingshot-1L (SSH1L) in pancreatic cancer contributes to tumor cell migration [163]. This enzyme is activated by F-actin which is formed in high quantity during lamellipodial assembly in malignant cells [164]. Phosphorylation and inhibition of SSH1L by protein kinase D (PDK) suppress cancer cell migration [165]. The role of protein kinase LIMK1 in tumor invasion and metastasis is still under discussion, similarly to its role in cell migration. According to various authors, LIMK1 caused either a decrease [119,159] or an increase [122,166,167] in invasion and metastasis. In metastatic rat mammary adenocarcinoma cells, the expression of the kinase domain of LIMK1, resulting in the near total phosphorylation of cofilin, completely inhibited the appearance of barbed ends and lamellipodia protrusion in response to EGF stimulation [119]. Overexpression of LIMK1 suppressed EGF-induced membrane protrusion and locomotion in rat mammary carcinoma cells [159]. In contrast, the increased activity of LIMK1 led to human breast cancer progression [166]. The level and activity of endogenous LIMK1 was increased in invasive breast and prostate cancer cell lines in comparison with less invasive cells [167]. The knockdown of LIMK1 has suppressed chemokine-induced lamellipodium formation and migration of Jurkat T cells [122]. These data about the positive role of LIMK1 in tumor cell migration at first seem to contradict the mechanism of tumor progression related to Cfl-1 dephosphorylation. Thus, some researchers have suggested that LIMK1 may play a role in regulating tumor progression via other mechanisms, independent of cofilin. For example, Bagheri-Yarmand et al. proposed that LIMK1 increases tumor metastasis of human breast cancer cells through stimulation of urokinase-type plasminogen activator system and degradation of the extracellular matrix by the serine protease urokinase type plasminogen activator [168]. However, there is a body of evidence that LIMK1 can influence the metastatic phenotype of tumor cells via regulation of cofilin activity, and the controversial effects of LIMK1 expression on migration and metastasis of cancer cells require an explanation. Wang et al. suggested that LIMK1 expression alone does not determine the motility and invasion status of carcinoma cells, and the collective activity and the output (barbed end production) of the LIMK1/cofilin pathways should be estimated [159]. Besides that, the contradictory results from different groups may be caused by different cell types used in these studies. 

It has been shown that oncoproteins and tumor suppressor proteins have effect on invasive and metastatic potential of tumors through cofilin-regulating pathways. One of the most known oncoproteins, tyrosine-protein kinase transforming protein of Rous sarcoma virus (v-Src) can disrupt the functioning of the Rho–ROCK–LIM kinase pathway resulting in dephosporylation of Cfl-1 and increased level of active Cfl-1 [169]. The tumor suppressor protein phosphoinositide phosphatase and tensin homolog (PTEN) may inactivate cofilin in cancer cells, while loss of PTEN and activation of phosphoinositide 3-kinase (PI3K) caused differential activation of the cofilin regulators, LIMK1 and SSH1L, and cofilin dephosphorylation, that promote microtentacles formation and enhance metastatic risk [170]. In addition, it was shown that the tumor suppressor Ras association domain-containing protein 1 (RASSF1A) blocks tumor growth by stimulating cofilin/PP2A-mediated dephosphorylation [161].

**Figure 5:**
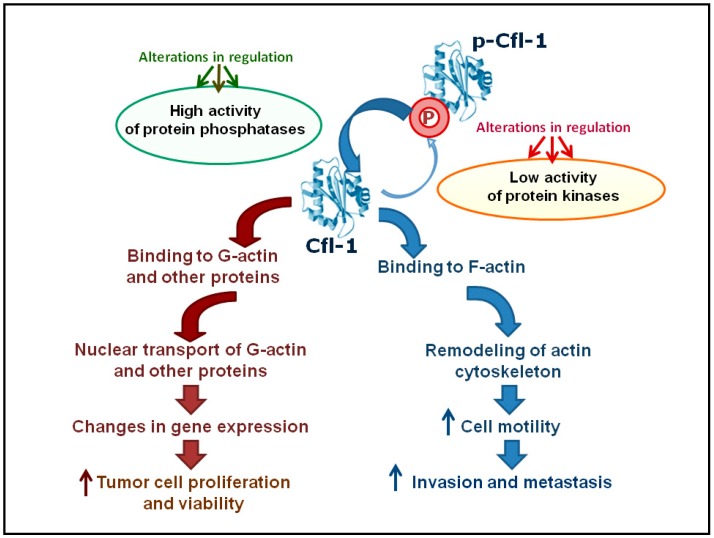
Simplified scheme showing changes in Cfl-1 functions in HMCs. This scheme does not consider, for example, the possible positive role of LIMK1 overexpression in invasion and metastasis. Alterations in regulation (green) and alterations in regulation (red) refer to changes in signaling pathways that lead to increased Cfl-1 dephosphorylation (activation) or decreased phosphorylation (inactivation), respectively.

Thus, the role of Cfl-1 as an important participant of various signaling pathways in HMCs requires further investigation. The contributions of Cfl-1 to the malignant phenotype are schematically presented in Figure 5. Obviously, the results of its study might be interesting in designing new approaches to early diagnostics and to rational treatment.

There are few publications about the presence of Cfl-2 in HMCs and cancer tissues. The muscle isoform of Cfl-2 (Cfl-2b) is considered as a biomarker of muscle differentiation [171] and has been identified in high quantity in well-differentiated leiomyosarcomas compared to undifferentiated pleomorphic sarcomas [172]. The expression level of Cfl-2 has prognostic significance in primary leiomyosarcomas independent of the histopathological type of tumor, and its expression correlates with improved disease-specific survival [172]. Cfl-2 has also been identified in HMCs of non-muscle origin [173,174,175,176]. Cfl-2 has been overexpressed in aggressive breast cancer cell lines, and its expression has been correlated with tumor grade in primary breast cancer tissue [175]. Significant upregulation of Cfl-1 and downregulation of Cfl-2 has been observed in pancreatic adenocarcinomas compared to non-cancerous tissues [173]. Dstn is the third traditional member of ADF/cofilins family that has also been identified in HMCs, mainly in different adenocarcinomas [6,18,143,177]. Likewise Cfl-1, Dstn can be a potentional biomarker of resistance to platinum-based agents [143]. The structural and functional similarities of traditional cofilins (in particular, the ability to undergo phosphorylation-dephosphorylation on Ser3) suggest that Cfl-1, Cfl-2 and Dstn may also be involved in the same pathways. Overexpression of LIMK1, Cfl-1, and Cfl-2 has been associated with low expression of mitogen-activated protein kinase MAPK1 (which is involved in cell growth and proliferation), and with enhanced survival of the patients with glioblastoma multiforme [174]. Dstn like Cfl-1 promotes tumor cell migration and invasiveness, but in general the activities of Dstn and Cfl-1 are non-overlapping [6,177].

### 3.2. Other Actin-Depolymerizing Factor/Cofilin Superfamily Proteins

Data on the expression of twinfilins in HMCs have been initially obtained using transcriptomics approaches. It has been shown that twinfilin might be a key determinant of lymphoma progression through regulation of actin dynamics. Moreover, twinfilin suppressed the action of the front-line chemotherapeutic agent vincristine in Eµ-myc lymphoma cells [178]. In prostate cancer cells, an osteoblast master transcription factor Runx2 is aberrantly expressed and promotes metastatic phenotype of cells through up-regulation of twinfilin gene and other genes with cancer associated functions [179]. TWF1 has been detected as a target for microRNA-206 (miR-206) which is referred to microRNAs, fundamental post-transcriptional regulators inhibiting gene expression. Blocking TWF1 by miR-206 in human xenograft models of breast cancer can suppress tumor invasion and metastasis by inhibiting the actin cytoskeleton dynamics [180]. 

Drebrins are considered as brain-specific intracellular regulators of morphogenesis [36,181]. The first report on drebrin detection in HMCs was published by Asada et al., who detected drebrin (namely, drebrin E2) in cultured neuroblastoma cells [182]. Later, data on the presence of drebrins in non-neuronal tumor tissues, especially in gliomas and malignant epithelial tumors, were published. The level of this protein in glioma cell lines varies and is equivalent or higher in comparison with the normal cells [183]. High expression level of this protein in glioma U87 cells transfected with a drebrin expression construct induces increased invasiveness and provides cell motility. On the contrary, knockdown of *DBN1* in glioma cells by small interfering RNA (siRNA) leads to decrease of cell migration and invasiveness [183]. It has been demonstrated that basal cell carcinomas are rich in drebrin, while keratinocytes of normal epidermis contain almost no drebrin, and that drebrin has potential value in diagnosis of basal cell carcinomas [98]. Drebrin has also been assumed as a potential biomarker for bladder cancer [184]. This protein can be considered a prognostic marker in patients with small lung cancer [185]. Proteomic analysis of colorectal cancer cell lines revealed drebrin to be overexpressed during liver metastasis [186]. The exact role of drebrin in epithelial tumor growth and formation of invasive and metastatic cell phenotype is still unclear. In urothelial carcinoma cell lines, drebrin has been shown to be critical for progranulin-dependent activation of the Akt and MAPK pathways and to modulate motility, invasion and anchorage-independent growth of tumor [184]. The drebrin-like protein (synonyms: mAbp1, HIP-55) known as the mammalian homologue of the yeast Abp1 has been poorly studied in HMCs, and the studies provide contradictory results. It was found that mAbp1 was upregulated or downregulated in several types of tumor tissues, and the highest expression was shown in lung cancer tissues. This protein increased the viability and decreased the apoptosis of lung cancer A549 cells treated with the anticancer agent etoposide [187]. It has been also reported that mAbp1 interacts with transcription regulator FHL-2 (four and a half LIM domains protein 2) and participates in negative regulation of Rho signaling and breast cancer cell invasion [188].

The GMF-B protein was initially characterized as a protein of neural tissue of vertebrates, which is able to affect the growth of normal and malignant glial cells in vitro and in vivo [41,42,189]. The molecular effects of GMF-B on HMCs of neuronal origin are diverse and contribute to contradictory results of studies. In particular, this protein was shown to stimulate DNA synthesis and proliferation of glioma cells and hybrid cells derived from glioma and neuroblastoma (NG108-15) cells, but had no effect on neuroblastoma cells [189]. In glioma cell lines of rodent and human origin, GMF-B promoted the initial growth of cell lines, but limited the proliferation by contact inhibition at the next steps [42]. In rat glioma cells, after transfection with GMF-B the enhanced expression of neurotrophic factors including nuclear factor-κ B was detected [190]. These results suggest a cytoprotective role for endogenous GMF in glial cells. In parallel, GMF-B was demonstrated to cause glioma progression via promoting neovascularization [191]. Finally, it was found that induced overexpression of GMF-B protein in neuroblastoma cells caused the cytotoxicity and loss viability via activation of glycogen synthase kinase-3β and caspase-3 [192]. GMF-B has been also found in non-brain tumors. Screening using retroviral expression libraries allowed detection of GMF-B encoding gene among genes involved in ovarian carcinogenesis [193]. The GMF-B protein was significantly overexpressed in serous ovarian carcinoma compared to normal epithelium, benign serous adenoma and borderline serous adenoma tissues, and high expression of GMF-B was associated with poor disease-free survival and overall survival [194]. There is only one report about identification of GMF-G in HMCs. Recently, Zuo et al. showed that the high GMF-G expression correlates with poor prognosis and promotes cell migration and invasion in epithelial ovarian cancer [195].

Human coactosin-like protein (COTL-1) has not been very actively studied in HMCs. The first reports on identification of COTL-1 in HMCs were published by Nakatsura et al. [196]. COTL-1 was detected by the serological expression cloning method (SEREX) in human pancreatic adenocarcinoma cell lines among a number of other pancreatic cancer antigens. The authors assumed that peptides from COTL-1 might be appropriate vaccine candidates for peptide-based immunotherapy of prostate cancer patients [196]. Later, proteomic analysis of PaCa44 pancreatic adenocarcinoma cell line treated with a chemoterapeutic agent, 5-aza-2′-deoxycytidine (DAC), revealed the 22-fold decreased expression of COTL-1 along with silence of cofilin and profilin 1 [197]. After that, Oh et al. using 2-DE with mass spectrometric identification revealed COTL-1 as a differentiation-related cytoskeleton protein in neuroblastoma cells [198]. Hou et al. also reported detection of COTL-1 in N1E-115 neuroblastoma cells [106]. Moreover, COTL-1 may also present in poorly differentiated cells, for example, in the case of high aggressive small cell lung cancer [199]. The comparison of small cell lung cancer tissues with normal bronchial epithelium showed more than 2-fold upregulation of this protein in cancer specimens. COTL-1 was immunohistochemically detected in 93% of small cell lung cancer tissue specimens and only in 16% of non-small cell lung cancer samples. On this basis authors assumed that COTL-1 may be a biomarker or a therapeutic target for patients with small cell lung cancer [199].

To sum up, despite the definite role in tumors, the mechanisms involving twinfilins, drebrin and drebrin-like protein, GMFs, and coactosin-like protein in malignant phenotype are still unclear. Consequently, new studies are needed to clarify their roles in tumors.

## 4. Conclusions

Cofilin-1 is found in all vertebrates and in many other organisms and plays an essential role in actin filament dynamics and reorganization through severing actin filaments. This function of Cfl-1 is regulated by several mechanisms including phosphorylation on Ser3. Active (dephosphorylated) Cfl-1, in addition to the main function, is able to provide transport of G-actin and some other proteins to the nucleus which is accompanied by changes in gene expression. Phospho-Cfl-1, considered by many authors as inactive, has been found to have its own function, namely, direct activation of phospholipase D1. Thus, Cfl-1 can be considered as a multifunctional protein which is involved in several signaling pathways regulating cell motility and development. HMCs of different origin contain Cfl-1 as one of the most abundant proteins. The expression level of Cfl-1 is often increased in HMCs, which underlines its contribution to malignant phenotype. There are several mechanisms involving Cfl-1 in tumor proliferation, invasion, and metastasis that are realized mainly through changes in the balance of kinases, phosphatases, and other proteins involved in cofilin-regulating pathways. Cofilin phosphatase SSH1 has been found to be upregulated in various invasive cancer cells. Cofilin kinase LIMK1 has also shown to play a pivotal role in cell motility. However, some studies provide contradictory data concerning the influence of the expression level of LIMK1 on cell migration, invasion, and metastasis.

The characteristic structural feature of Cfl-1 is the presence of special ADF-H domain in its structure. The ADF-H domains have also been identified in a number of other proteins that can directly or indirectly interact with actin cytoskeleton and provide its remodeling. These proteins, differing in size and functionality, are currently referred to as ADF/cofilin superfamily. Almost all of these proteins are direct or indirect regulators of cell motility. In addition, drebrin, drebrin-like protein, and glia maturation factors are characterized as regulators of cellular differentiation. Therefore, all ADF/cofilin superfamily members can contribute to malignant phenotypes of HMCs. However, available data on the functions and presence of many ADF/cofilin superfamily proteins in HMCs are still limited and conflicting. For instance, conflicting results were obtained concerning the role of mAbp1 and GMF-B in invasion and metastasis.

The controversial data on the role of dephosphorylated Cfl-1, LIMK1, mAbp1, and GMF-B in cell motility, invasion and metastasis may have several possible reasons including different cell types used in the studies, intratumoral cell heterogeneity, distinct functions of studied proteins at different stages of development or tumor progression, cellular background, etc. All these factors should be taken into account, and the collective activity of cofilin-regulating pathways should be estimated for evaluation of the invasive and metastatic potential of HMCs. With due consideration of these factors, further studies of ADF/cofilin superfamily proteins in HMCs can be a very promising research direction, which may extend the understanding of the molecular basis of tumor phenotypes and provide new protein targets for molecular and clinical oncology.

## Figures and Tables

**Table 1:** Characteristics of the main human actin-depolymerizing factor (ADF)/cofilin superfamily members according to [56,57,58,59,60,61,62,63] and UniProt.

Protein Name, *Gene Symbol* (UniProt Number)	Length (Positions *)	Domains, Length (Positions)	Motifs, Signals	Binding Partners
Cofilin-1, *CFL1* (P23528)	165 * (2–166)	ADF-H, 150 (4–153)	Nuclear localization signal	F- and G-actin (actin depolymerization and polymerization), PIP2, cortactin, LIMK1
Cofilin-2, *CFL2* ** (Q9Y281)	165 * (2–166)	ADF-H, 150 (4–153)	Nuclear localization signal	F- and G-actin (actin depolymerization and polymerization), PIP2
Destrin, *DSTN* (P60981)	164 * (2–165)	ADF-H, 150 (4–153)	Nuclear localization signal	F- and G-actin (actin depolymerization and polymerization), PIP2
Twinfilin-1, *TWF1* ** (Q12792)	349 * (2–350)	ADF-H 1, 138 (2–139)		G-actin and F-actin (barbed-end-capping activity)
		ADF-H 2, 139 (175–313)		
Twinfilin-2, *TWF2* (Q6IBS0)	348 * (2–349)	ADF-H 1, 136 (4–139)		G-actin and F-actin (barbed-end-capping activity)
		ADF-H 2, 137 (177–313)		
Drebrin, *DBN1* ** (Q16643)	648 * (2–649)	ADF-H, 130 (3–134)	Proline-rich, profilin-binding motif	F-actin (actin stabilization), cyclin-dependent kinase 5, connexin 43 and other proteins
Drebrin-like protein, *DBNL* ** (Q9UJU6)	430 (1–430)	ADF-H, 130 (4–133)	Proline-rich, Ser269/Thr291-phospho-sensor motif	F-actin (actin stabilization), SH3 and multiple ankyrin repeat domains proteins
		SH3, 60 (371–430)		
Glia maturation factor beta, *GMFB* (P60983)	141 * (2–142)	ADF-H, 136 (4–139)		Proteins of Arp2/3 complex (actin disassembly)
Glia maturation factor gamma, *GMFG* (O60234)	141 * (2–142)	ADF-H, 136 (4–139)		F-actin, proteins of Arp2/3 complex (actin disassembly)
Coactosin-like protein, *COTL1* (Q14019)	141 * (2–142)	ADF-H, 129 (2–130)		F-actin (actin stabilization)

* After removing initiator methionine; ** Expression with alternative splicing and formation of different transcripts. PIP2: phosphatidylinositol 4,5-bisphosphate; LIMK1: LIM domain kinase 1; F-actin: filamentous actin; G-actin: globular actin; SH3: Src homology 3 domain; Arp2/3: Actin-related protein 2/3 complex.

## References

[1] Sell S., Pierce G.B. (1994). Maturation arrest of stem cell differentiation is a common pathway for the cellular origin of teratocarcinomas and epithelial cancers. Lab. Investig..

[2] Hardavella G., George R., Sethi T. (2016). Lung cancer stem cells-characteristics, phenotype. Transl. Lung Cancer Res..

[3] Paszek M.J., Zahir N., Johnson K.R., Lakins J.N., Rozenberg G.I., Gefen A., Reinhart-King C.A., Margulies S.S., Dembo M., Boettiger D. (2005). Tensional homeostasis and the malignant phenotype. Cancer Cell.

[4] Boja E.S., Rodriguez H. (2014). Proteogenomic convergence for understanding cancer pathways and networks. Clin. Proteom..

[5] Carpenter R.L., Lo H.W. (2012). Hedgehog pathway and GLI1 isoforms in human cancer. Discov. Med..

[6] Tahtamouni L.H., Shaw A.E., Hasan M.H., Yasin S.R., Bamburg J.R. (2013). Non-overlapping activities of ADF and cofilin-1 during the migration of metastatic breast tumor cells. BMC Cell Biol..

[7] Kojima M., Higuchi Y., Yokota M., Ishii G., Saito N., Aoyagi K., Sasaki H., Ochiai A. (2014). Human subperitoneal fibroblast and cancer cell interaction creates microenvironment that enhances tumor progression and metastasis. PLoS ONE.

[8] Bravo-Cordero J.J., Hodgson L., Condeelis J.S. (2014). Spatial regulation of tumor cell protrusions by RhoC. Cell Adhes. Migr..

[9] Martin S.K., Kamelgarn M., Kyprianou N. (2014). Cytoskeleton targeting value in prostate cancer treatment. Am. J. Clin. Exp. Urol..

[10] Weaver A.M. (2008). Cortactin in tumor invasiveness. Cancer Lett..

[11] Albiges-Rizo C., Destaing O., Fourcade B., Planus E., Block M.R. (2009). Actin machinery and mechanosensitivity in invadopodia, podosomes and focal adhesions. J. Cell Sci..

[12] Madsen C.D., Hooper S., Tozluoglu M., Bruckbauer A., Fletcher G., Erler J.T., Bates P.A., Thompson B., Sahai E. (2015). STRIPAK components determine mode of cancer cell migration and metastasis. Nat. Cell Biol..

[13] Gau D.M., Lesnock J.L., Hood B.L., Bhargava R., Sun M., Darcy K., Luthra S., Chandran U., Conrads T.P., Edwards R.P. (2015). BRCA1 deficiency in ovarian cancer is associated with alteration in expression of several key regulators of cell motility—A proteomics study. Cell Cycle.

[14] Wang W., Eddy R., Condeelis J. (2007). The cofilin pathway in breast cancer invasion and metastasis. Nat. Rev. Cancer.

[15] Shishkin S., Kovaleva M., Ivanov A., Eryomina L., Lisitskaya K., Toropugin I., Kovalev L., Okhritz V., Loran O. (2011). Comparative proteomic study of proteins in prostate cancer and benign hyperplasia cells. J. Cancer Sci. Ther..

[16] Huang L., Kuwahara I., Matsumoto K. (2014). EWS represses cofilin 1 expression by inducing nuclear retention of cofilin 1 mRNA. Oncogene.

[17] Chang C.Y., Leu J.D., Lee Y.J. (2015). The actin depolymerizing factor (ADF)/cofilin signaling pathway and DNA damage responses in cancer. Int. J. Mol. Sci..

[18] Estornes Y., Gay F., Gevrey J.C., Navoizat S., Nejjari M., Scoazec J.Y., Chayvialle J.A., Saurin J.C., Abello J. (2007). Differential involvement of destrin and cofilin-1 in the control of invasive properties of Isreco1 human colon cancer cells. Int. J. Cancer.

[19] Bockhorn J., Yee K., Chang Y.F., Prat A., Huo D., Nwachukwu C., Dalton R., Huang S., Swanson K.E., Perou C.M. (2013). MicroRNA-30c targets cytoskeleton genes involved in breast cancer cell invasion. Breast Cancer Res. Treat..

[20] Multi-Level Information Database “Proteomics of Prostate Cancer”. http://ef.inbi.ras.ru.

[21] Multi-Level Information Database “Proteomics of Malignant Cells”. http://ef2.inbi.ras.ru.

[22] Bamburg J.R., Harris H.E., Weeds A.G. (1980). Partial purification and characterization of an actin depolymerizing factor from brain. FEBS Lett..

[23] Thorstensson R., Sterky C., Norberg R. (1985). Preparation of highly purified F-actin-depolymerizing factor of human serum. Eur. J. Biochem..

[24] Nishida E., Maekawa S., Sakai H. (1984). Cofilin, a protein in porcine brain that binds to actin filaments and inhibits their interactions with myosin and tropomyosin. Biochemistry.

[25] Vartiainen M.K., Mustonen T., Mattila P.K., Ojala P.J., Thesleff I., Partanen J., Lappalainen P. (2002). The three mouse actin-depolymerizing factor/cofilins evolved to fulfill cell-type-specific requirements for actin dynamics. Mol. Biol. Cell.

[26] Li G.H., Arora P.D., Chen Y., McCulloch C.A., Liu P. (2012). Multifunctional roles of gelsolin in health and diseases. Med. Res. Rev..

[27] Moon A., Drubin D.G. (1995). The ADF/cofilin proteins: stimulus-responsive modulators of actin dynamics. Mol. Biol. Cell.

[28] Ono S., Baillie D.L., Benian G.M. (1999). UNC-60B, an ADF/cofilin family protein, is required for proper assembly of actin into myofibrils in *Caenorhabditis elegans* body wall muscle. J. Cell Biol..

[29] Lappalainen P., Kessels M.M., Cope M.J., Drubin D.G. (1998). The ADF homology (ADF-H) domain: A highly exploited actin-binding module. Mol. Biol. Cell.

[30] PDBsum: Pictorial Database of 3D Structures in the Protein Data Bank. http://www.ebi.ac.uk/thornton-srv/databases/pdbsum.

[31] Takagi T., Konishi K., Mabuchi I. (1988). Amino acid sequence of starfish oocyte depactin. J. Biol. Chem..

[32] De Hostos E.L., Bradtke B., Lottspeich F., Gerisch G. (1993). Coactosin, a 17 kDa F-actin binding protein from *Dictyostelium discoideum*. Cell Motil. Cytoskelet..

[33] Leonard S., Gittis A., Petrella E., Pollard T., Lattman E. (1997). Crystal structure of the actin-binding protein actophorin from *Acanthamoeba*. Nat. Struct. Biol..

[34] Goode B.L., Drubin D.G., Lappalainen P. (1998). Regulation of the cortical actin cytoskeleton in budding yeast by twinfilin, a ubiquitous actin monomer-sequestering protein. J. Cell Biol..

[35] Park H., Yamada K., Kojo A., Sato S., Onozuka M., Yamamoto T. (2009). Drebrin (developmentally regulated brain protein) is associated with axo-somatic synapses and neuronal gap junctions in rat mesencephalic trigeminal nucleus. Neurosci. Lett..

[36] Shirao T. (1995). The roles of microfilament-associated proteins, drebrins, in brain morphogenesis: A review. J. Biochem..

[37] Goode B.L., Rodal A.A., Barnes G., Drubin D.G. (2001). Activation of the Arp2/3 complex by the actin filament binding protein Abp1p. J. Cell Biol..

[38] Le Bras S., Foucault I., Foussat A., Brignone C., Acuto O., Deckert M. (2004). Recruitment of the actin-binding protein HIP-55 to the immunological synapse regulates T cell receptor signaling and endocytosis. J. Biol. Chem..

[39] Goroncy A.K., Koshiba S., Tochio N., Tomizawa T., Sato M., Inoue M., Watanabe S., Hayashizaki Y., Tanaka A., Kigawa T. (2009). NMR solution structures of actin depolymerizing factor homology domains. Protein Sci..

[40] Nakano K., Kuwayama H., Kawasaki M., Numata O., Takaine M. (2010). GMF is an evolutionarily developed Adf/cofilin-super family protein involved in the Arp2/3 complex-mediated organization of the actin cytoskeleton. Cytoskeleton.

[41] Lim R., Nakagawa S., Arnason B.G., Turriff D.E. (1981). Glia maturation factor promotes contact inhibition in cancer cells. Proc. Natl. Acad. Sci. USA.

[42] Lim R., Hicklin D.J., Ryken T.C., Han X.M., Liu K.N., Miller J.F., Baggenstoss B.A. (1986). Suppression of glioma growth in vitro and in vivo by glia maturation factor. Cancer Res..

[43] Thirion C., Stucka R., Mendel B., Gruhler A., Jaksch M., Nowak K.J., Binz N., Laing N.G., Lochmüller H. (2001). Characterization of human muscle type cofilin (CFL2) in normal and regenerating muscle. Eur. J. Biochem..

[44] Bravo-Cordero J.J., Magalhaes M.A., Eddy R.J., Hodgson L., Condeelis J. (2013). Functions of cofilin in cell locomotion and invasion. Nat. Rev. Mol. Cell Biol..

[45] Shukla V.K., Kabra A., Maheshwari D., Yadav R., Jain A., Tripathi S., Ono S., Kumar D., Arora A. (2015). Solution structures and dynamics of ADF/cofilins UNC-60A and UNC-60B from *Caenorhabditis elegans*. Biochem. J..

[46] Carlier M.F., Laurent V., Santolini J., Melki R., Didry D., Xia G.X., Hong Y., Chua N.H., Pantaloni D. (1997). Actin depolymerizing factor (ADF/cofilin) enhances the rate of filament turnover: Implication in actin-based motility. J. Cell Biol..

[47] Yonezawa N., Homma Y., Yahara I., Sakai H., Nishida E. (1991). A short sequence responsible for both phosphoinositide binding and actin binding activities of cofilin. J. Biol. Chem..

[48] Ivanovska J., Tregubova A., Mahadevan V., Chakilam S., Gandesiri M., Benderska N., Ettle B., Hartmann A., Söder S., Ziesché E. (2013). Identification of DAPK as a scaffold protein for the LIMK/cofilin complex in TNF-induced apoptosis. Int. J. Biochem. Cell Biol..

[49] Nevalainen E.M., Skwarek-Maruszewska A., Braun A., Moser M., Lappalainen P. (2009). Two biochemically distinct and tissue-specific twinfilin isoforms are generated from the mouse Twf2 gene by alternative promoter usage. Biochem. J..

[50] Poukkula M., Kremneva E., Serlachius M., Lappalainen P. (2011). Actin-depolymerizing factor homology domain: A conserved fold performing diverse roles in cytoskeletal dynamics. Cytoskeleton.

[51] Pérez-Martínez M., Gordón-Alonso M., Cabrero J.R., Barrero-Villar M., Rey M., Mittelbrunn M., Lamana A., Morlino G., Calabia C., Yamazaki H. (2010). F-actin-binding protein drebrin regulates CXCR4 recruitment to the immune synapse. J. Cell Sci..

[52] Ikeda K., Kundu R.K., Ikeda S., Kobara M., Matsubara H., Quertermous T. (2006). Glia maturation factor-gamma is preferentially expressed in microvascular endothelial and inflammatory cells and modulates actin cytoskeleton reorganization. Circ. Res..

[53] Lippert D.N., Wilkins J.A. (2012). Glia maturation factor gamma regulates the migration and adherence of human T lymphocytes. BMC Immunol..

[54] Kim J., Shapiro M.J., Bamidele A.O., Gurel P., Thapa P., Higgs H., Hedin K.E., Shapiro V.S., Billadeau D.D. (2014). Coactosin-like 1 antagonizes cofilin to promote lamellipodial protrusion at the immune synapse. PLoS ONE.

[55] Kumar N., Somlata, Mazumder M., Dutta P., Maiti S., Gourinath S. (2014). EhCoactosin stabilizes actin filaments in the protist parasite *Entamoeba histolytica*. PLoS Pathog..

[56] Paavilainen V.O., Hellman M., Helfer E., Bovellan M., Annila A., Carlier M.F., Permi P., Lappalainen P. (2007). Structural basis and evolutionary origin of actin filament capping by twinfilin. Proc. Natl. Acad. Sci. USA.

[57] Takacs-Kollar V., Nyitrai M., Hild G. (2016). The effect of mouse twinfilin-1 on the structure and dynamics of monomeric actin. Biochim. Biophys. Acta.

[58] Peitsch W.K., Hofmann I., Prätzel S., Grund C., Kuhn C., Moll I., Langbein L., Franke W.W. (2001). Drebrin particles: Components in the ensemble of proteins regulating actin dynamics of lamellipodia and filopodia. Eur. J. Cell Biol..

[59] Worth D.C., Daly C.N., Geraldo S., Oozeer F., Gordon-Weeks P.R. (2013). Drebrin contains a cryptic F-actin-bundling activity regulated by Cdk5 phosphorylation. J. Cell Biol..

[60] Gordon-Weeks P.R. (2016). The role of the drebrin/EB3/Cdk5 pathway in dendritic spine plasticity, implications for Alzheimer’s disease. Brain Res. Bull..

[61] Ambrosi C., Ren C., Spagnol G., Cavin G., Cone A., Grintsevich E.E., Sosinsky G.E., Sorgen P.L. (2016). Connexin43 Forms Supramolecular Complexes through Non-Overlapping Binding Sites for Drebrin, Tubulin, and ZO-1. PLoS ONE.

[62] Cortesio C.L., Perrin B.J., Bennin D.A., Huttenlocher A. (2010). Actin-binding protein-1 interacts with WASp-interacting protein to regulate growth factor-induced dorsal ruffle formation. Mol. Biol. Cell.

[63] Li Z., Park H.R., Shi Z., Li Z., Pham C.D., Du Y., Khuri F.R., Zhang Y., Han Q., Fu H. (2014). Pro-oncogenic function of HIP-55/Drebrin-like (DBNL) through Ser269/Thr291-phospho-sensor motifs. Oncotarget.

[64] Andrianantoandro E., Pollard T.D. (2006). Mechanism of actin filament turnover by severing and nucleation at different concentrations of ADF/cofilin. Mol. Cell.

[65] Dos Remedios C.G., Chhabra D., Kekic M., Dedova I.V., Tsubakihara M., Berry D.A., Nosworthy N.J. (2003). Actin Binding Proteins: Regulation of Cytoskeletal Microfilaments. Physiol. Rev..

[66] Winder S.J., Аyscough K.R. (2005). Actin-binding proteins. J. Cell Sci..

[67] Tania N., Condeelis J., Edelstein-Keshet L. (2013). Modeling the synergy of cofilin and Arp2/3 in lamellipodial protrusive activity. Biophys. J..

[68] Chan C., Beltzner C.C., Pollard T.D. (2009). Cofilin dissociates Arp2/3 complex and branches from actin filaments. Curr. Biol..

[69] Nakashima K., Sato N., Nakagaki T., Abe H., Ono S., Obinata T. (2005). Two mouse cofilin isoforms, muscle-type (MCF) and non-muscle type (NMCF), interact with F-actin with different efficiencies. J. Biochem..

[70] Yahara I., Aizawa H., Moriyama K., Iida K., Yonezawa N., Nishida E., Hatanaka H., Inagaki F. (1996). A role of cofilin/destrin in reorganization of actin cytoskeleton in response to stresses and cell stimuli. Cell Struct. Funct..

[71] Tojkander S., Gateva G., Lappalainen P. (2012). Actin stress fibers—Assembly, dynamics and biological roles. J. Cell Sci..

[72] Kuure S., Cebrian C., Machingo Q., Lu B.C., Chi X., Hyink D., D’agati V., Gurniak C., Witke W., Costantini F. (2010). Actin depolymerizing factors cofilin1 and destrin are required for ureteric bud branching morphogenesis. PLoS Genet..

[73] Sparrow N., Manetti M.E., Bott M., Fabianac T., Petrilli A., Bates M.L., Bunge M.B., Lambert S., Fernandez-Valle C. (2012). The actin-severing protein cofilin is downstream of neuregulin signaling and is essential for Schwann cell myelination. J. Neurosci..

[74] Dumpich M., Mannherz H.G., Theiss C. (2015). VEGF Signaling Regulates Cofilin and the Arp2/3-complex within the Axonal Growth Cone. Curr. Neurovasc. Res..

[75] Aragona M., Panciera T., Manfrin A., Giulitti S., Michielin F., Elvassore N., Dupont S., Piccolo S. (2013). A mechanical checkpoint controls multicellular growth through YAP/TAZ regulation by actin-processing factors. Cell.

[76] Ohashi K. (2015). Roles of cofilin in development and its mechanisms of regulation. Dev. Growth Differ..

[77] Nebl G., Meuer S.C., Samstag Y. (1996). Dephosphorylation of serine 3 regulates nuclear translocation of cofilin. J. Biol. Chem..

[78] Ono S., Abe H., Nagaoka R., Obinata T. (1993). Colocalization of ADF and cofilin in intranuclear actin rods of cultured muscle cells. J. Muscle Res. Cell Motil..

[79] Percipalle P. (2013). Co-transcriptional nuclear actin dynamics. Nucleus.

[80] Samstag Y., Eckerskorn C., Wesselborg S., Henning S., Wallich R., Meuer S.C. (1994). Costimulatory signals for human T-cell activation induce nuclear translocation of pp19/cofilin. Proc. Natl. Acad. Sci. USA.

[81] Lee K.H., Meuer S.C., Samstag Y. (2000). Cofilin: A missing link between T cell co-stimulation and rearrangement of the actin cytoskeleton. Eur. J. Immunol..

[82] Samstag Y., Dreizler E.M., Ambach A., Sczakiel G., Meuer S.C. (1996). Inhibition of constitutive serine phosphatase activity in T lymphoma cells results in phosphorylation of pp19/cofilin and induces apoptosis. J. Immunol..

[83] Sen B., Xie Z., Uzer G., Thompson W.R., Styner M., Wu X., Rubin J. (2015). Intranuclear Actin Regulates Osteogenesis. Stem Cells.

[84] Klamt F., Zdanov S., Levine R.L., Pariser A., Zhang Y., Zhang B., Yu L.R., Veenstra T.D., Shacter E. (2009). Oxidant-induced apoptosis is mediated by oxidation of the actin-regulatory protein cofilin. Nat. Cell Biol..

[85] Han L., Stope M.B., de Jesús M.L., Oude Weernink P.A., Urban M., Wieland T., Rosskopf D., Mizuno K., Jakobs K.H., Schmidt M. (2007). Direct stimulation of receptor-controlled phospholipase D1 by phospho-cofilin. EMBO J..

[86] Gomez-Cambronero J. (2014). Phospholipase D in cell signaling: from a myriad of cell functions to cancer growth and metastasis. J. Biol. Chem..

[87] Gurniak C.B., Perlas E., Witke W. (2005). The actin depolymerizing factor n-cofilin is essential for neural tube morphogenesis and neural crest cell migration. Dev. Biol..

[88] Bellenchi G.C., Gurniak C.B., Perlas E., Middei S., Ammassari-Teule M., Witke W. (2007). N-cofilin is associated with neuronal migration disorders and cell cycle control in the cerebral cortex. Genes Dev..

[89] Gurniak C.B., Chevessier F., Jokwitz M., Jonsson F., Perlas E., Richter H., Matern G., Boyl P.P., Chaponnier C., Furst D. (2014). Severe protein aggregate myopathy in a knockout mouse model points to an essential role of cofilin2 in sarcomeric actin exchange and muscle maintenance. Eur. J. Cell Biol..

[90] Kanellos G., Frame M.C. (2016). Cellular functions of the ADF/cofilin family at a glance. J. Cell Sci..

[91] Vartiainen M.K., Sarkkinen E.M., Matilainen T., Salminen M., Lappalainen P. (2003). Mammals have two twinfilin isoforms whose subcellular localizations and tissue distributions are differentially regulated. J. Biol. Chem..

[92] Nevalainen E.M., Braun A., Vartiainen M.K., Serlachius M., Andersson L.C., Moser M., Lappalainen P. (2011). Twinfilin-2a is dispensable for mouse development. PLoS ONE.

[93] Hayashi K., Shirao T. (1999). Change in the shape of dendritic spines caused by overexpression of drebrin in cultured cortical neurons. J. Neurosci..

[94] Mizui T., Kojima N., Yamazaki H., Katayama M., Hanamura K., Shirao T. (2009). Drebrin E is involved in the regulation of axonal growth through actin-myosin interactions. J. Neurochem..

[95] Sonego M., Oberoi M., Stoddart J., Gajendra S., Hendricusdottir R., Oozeer F., Worth D.C., Hobbs C., Eickholt B.J., Gordon-Weeks P.R. (2015). Drebrin regulates neuroblast migration in the postnatal mammalian brain. PLoS ONE.

[96] Grintsevich E.E., Reisler E. (2014). Drebrin inhibits cofilin-induced severing of F-actin. Cytoskeleton.

[97] Keon B.H., Jedrzejewski P.T., Paul D.L., Goodenough D.A. (2000). Isoform specific expression of the neuronal F-actin binding protein, drebrin, in specialized cells of stomach and kidney epithelia. J. Cell Sci..

[98] Peitsch W.K., Hofmann I., Bulkescher J., Hergt M., Spring H., Bleyl U., Goerdt S., Franke W.W. (2005). Drebrin, an actin-binding, cell-type characteristic protein: Induction and localization in epithelial skin tumors and cultured keratinocytes. J. Investig. Dermatol..

[99] Mise-Omata S., Montagne B., Deckert M., Wienands J., Acuto O. (2003). Mammalian actin binding protein 1 is essential for endocytosis but not lamellipodia formation: Functional analysis by RNA interference. Biochem. Biophys. Res. Commun..

[100] Schymeinsky J., Sperandio M., Walzog B. (2011). The mammalian actin-binding protein 1 (mAbp1): a novel molecular player in leukocyte biology. Trends Cell. Bio..

[101] Gandhi M., Smith B.A., Bovellan M., Paavilainen V., Daugherty-Clarke K., Gelles J., Lappalainen P., Goode B.L. (2010). GMF is a cofilin homolog that binds Arp2/3 complex to stimulate filament debranching and inhibit actin nucleation. Curr. Biol..

[102] Poukkula M., Hakala M., Pentinmikko N., Sweeney M.O., Jansen S., Mattila J., Hietakangas V., Goode B.L., Lappalainen P. (2014). GMF promotes leading-edge dynamics and collective cell migration in vivo. Curr. Biol..

[103] Zaheer S., Thangavel R., Sahu S.K., Zaheer A. (2011). Augmented expression of glia maturation factor in Alzheimer’s disease. Neuroscience.

[104] Zaheer S., Thangavel R., Wu Y., Khan M.M., Kempuraj D., Zaheer A. (2013). Enhanced expression of glia maturation factor correlates with glial activation in the brain of triple transgenic Alzheimer’s disease mice. Neurochem. Res..

[105] Haynes E.M., Asokan S.B., King S.J., Johnson H.E., Haugh J.M., Bear J.E. (2015). GMFβ controls branched actin content and lamellipodial retraction in fibroblasts. J. Cell Biol..

[106] Hou X., Katahira T., Ohashi K., Mizuno K., Sugiyama S., Nakamura H. (2013). Coactosin accelerates cell dynamism by promoting actin polymerization. Dev. Biol..

[107] Hou X., Katahira T., Kimura J., Nakamura H. (2009). Expression of chick Coactosin in cells in morphogenetic movement. Dev. Growth Differ..

[108] Basavarajappa D., Wan M., Lukic A., Steinhilber D., Samuelsson B., Rådmark O. (2014). Roles of coactosin-like protein (CLP) and 5-lipoxygenase-activating protein (FLAP) in cellular leukotriene biosynthesis. Proc. Natl. Acad. Sci. USA.

[109] Yonezawa N., Nishida E., Sakai H. (1985). pH control of actin polymerization by cofilin. J. Biol. Chem..

[110] Bernstein B.W., Painter W.B., Chen H., Minamide L.S., Abe H., Bamburg J.R. (2000). Intracellular pH modulation of ADF/cofilin proteins. Cell Motil. Cytoskelet..

[111] Zhao H., Hakala M., Lappalainen P. (2010). ADF/cofilin binds phosphoinositides in a multivalent manner to act as a PIP(2)-density sensor. Biophys. J..

[112] Moriyama K., Iida K., Yahara I. (1996). Phosphorylation of Ser-3 of cofilin regulates its essential function on actin. Genes Cells.

[113] Agnew B.J., Minamide L.S., Bamburg J.R. (1995). Reactivation of phosphorylated actin depolymerizing factor and identification of the regulatory site. J. Biol. Chem..

[114] Bernstein B.W., Bamburg J.R. (2010). ADF/cofilin: A functional node in cell biology. Trends Cell Biol..

[115] Tomasella A., Blangy A., Brancolini C. (2014). A receptor-interacting protein 1 (RIP1)-independent necrotic death under the control of protein phosphatase PP2A that involves the reorganization of actin cytoskeleton and the action of cofilin-1. J. Biol. Chem..

[116] Abe H., Obinata T., Minamide L., Bamburg J.R. (1996). Xenopus laevis actin-depolymerizing factor/cofilin: A phosphorylation-regulated protein essential for development. J. Cell Biol..

[117] Obinata T., Nagaoka-Yasuda R., Ono S., Kusano K., Mohri K., Ohtaka Y., Yamashiro S., Okada K., Abe H. (1997). Low molecular-weight G-actin binding proteins involved in the regulation of actin assembly during myofibrillogenesis. Cell Struct. Funct..

[118] Davila M., Jhala D., Ghosh D., Grizzle W.E., Chakrabarti R. (2007). Expression of LIM kinase 1 is associated with reversible G1/S phase arrest, chromosomal instability and prostate cancer. Mol. Cancer.

[119] Zebda N., Bernard O., Bailly M., Welti S., Lawrence D.S., Condeelis J.S. (2000). Phosphorylation of ADF/cofilin abolishes EGF-induced actin nucleation at the leading edge and subsequent lamellipod extension. J. Cell Biol..

[120] Nishita M., Aizawa H., Mizuno K. (2002). Stromal cell-derived factor 1alpha activates LIM kinase 1 and induces cofilin phosphorylation for T-cell chemotaxis. Mol. Cell. Biol..

[121] Ghosh M., Song X., Mouneimne G., Sidani M., Lawrence D.S., Condeelis J.S. (2004). Cofilin promotes actin polymerization and defines the direction of cell motility. Science.

[122] Nishita M., Tomizawa C., Yamamoto M., Horita Y., Ohashi K., Mizuno K. (2005). Spatial and temporal regulation of cofilin activity by LIM kinase and Slingshot is critical for directional cell migration. J. Cell Biol..

[123] Mizuno K. (2013). Signaling mechanisms and functional roles of cofilin phosphorylation and dephosphorylation. Cell Signal..

[124] Beaty B.T., Condeelis J. (2014). Digging a little deeper: The stages of invadopodium formation and maturation. Eur. J. Cell Biol..

[125] Zalli D., Neff L., Nagano K., Shin N.Y., Witke W., Gori F., Baron R. (2016). The Actin-Binding Protein Cofilin and Its Interaction With Cortactin Are Required for Podosome Patterning in Osteoclasts and Bone Resorption In Vivo and In Vitro. Bone Miner. Res..

[126] Nomura K., Hayakawa K., Tatsumi H., Ono S. (2016). Actin-interacting Protein 1 Promotes Disassembly of Actin-depolymerizing Factor/Cofilin-bound Actin Filaments in a pH-dependent Manner. J. Biol. Chem..

[127] Zhou G.L., Zhang H., Field J. (2014). Mammalian CAP (Cyclase-associated protein) in the world of cell migration: Roles in actin filament dynamics and beyond. Cell Adhes. Migr..

[128] Mikati M.A., Breitsprecher D., Jansen S., Reisler E., Goode B.L. (2015). Coronin Enhances Actin Filament Severing by Recruiting Cofilin to Filament Sides and Altering F-Actin Conformation. J. Mol. Biol..

[129] Mouneimne G., Soon L., DesMarais V., Sidani M., Song X., Yip S.C., Ghosh M., Eddy R., Backer J.M., Condeelis J. (2004). Phospholipase C and cofilin are required for carcinoma cell directionality in response to EGF stimulation. J. Cell Biol..

[130] Chan A.Y., Bailly M., Zebda N., Segall J.E., Condeelis J.S. (2000). Role of cofilin in epidermal growth factor-stimulated actin polymerization and lamellipod protrusion. J. Cell Biol..

[131] Song X., Chen X., Yamaguchi H., Mouneimne G., Condeelis J.S., Eddy R.J. (2006). Initiation of cofilin activity in response to EGF is uncoupled from cofilin phosphorylation and dephosphorylation in carcinoma cells. J. Cell Sci..

[132] Fratelli M., Demol H., Puype M., Casagrande S., Eberini I., Salmona M., Bonetto V., Mengozzi M., Duffieux F., Miclet E. (2002). Identification by redox proteomics of glutathionylated proteins in oxidatively stressed human T lymphocytes. Proc. Natl. Acad. Sci. USA.

[133] Klemke M., Wabnitz G.H., Funke F., Funk B., Kirchgessner H., Samstag Y. (2008). Oxidation of cofilin mediates T cell hyporesponsiveness under oxidative stress conditions. Immunity.

[134] Zhang H.H., Wang W., Feng L., Yang Y., Zheng J., Huang L., Chen D.B. (2015). S-nitrosylation of Cofilin-1 Serves as a Novel Pathway for VEGF-Stimulated Endothelial Cell Migration. J. Cell. Physiol..

[135] Moseley J.B., Okada K., Balcer H.I., Kovar D.R., Pollard T.D., Goode B.L. (2006). Twinfilin is an actin-filament-severing protein and promotes rapid turnover of actin structures in vivo. J. Cell Sci..

[136] Tanabe K., Yamazaki H., Inaguma Y., Asada A., Kimura T., Takahashi J., Taoka M., Ohshima T., Furuichi T., Isobe T. (2014). Phosphorylation of drebrin by cyclin-dependent kinase 5 and its role in neuronal migration. PLoS ONE.

[137] Ketschek A., Spillane M., Dun X.P., Hardy H., Chilton J., Gallo G. (2016). Drebrin coordinates the actin and microtubule cytoskeleton during the initiation of axon collateral branches. Dev. Neurobiol..

[138] Wang T., Cleary R.A., Wang R., Tang D.D. (2014). Glia maturation factor-γ phosphorylation at Tyr-104 regulates actin dynamics and contraction in human airway smooth muscle. Am. J. Respir. Cell Mol. Biol..

[139] Stierum R., Gaspari M., Dommels Y., Ouatas T., Pluk H., Jespersen S., Vogels J., Verhoeckx K., Groten J., van Ommen B. (2003). Proteome analysis reveals novel proteins associated with proliferation and differentiation of the colorectal cancer cell line Caco-2. Biochim. Biophys. Acta.

[140] Zhang Y., Tong X. (2010). Expression of the actin-binding proteins indicates that cofilin and fascin are related to breast tumour size. J. Int. Med. Res..

[141] Neely B.A., Wilkins C.E., Marlow L.A., Malyarenko D., Kim Y., Ignatchenko A., Sasinowska H., Sasinowski M., Nyalwidhe J.O., Kislinger T. (2016). Proteotranscriptomic Analysis Reveals Stage Specific Changes in the Molecular Landscape of Clear-Cell Renal Cell Carcinoma. PLoS ONE.

[142] Wei R., Zhang Y., Shen L., Jiang W., Li C., Zhong M., Xie Y., Yang D., He L., Zhou Q. (2012). Comparative proteomic and radiobiological analyses in human lung adenocarcinoma cells. Mol. Cell. Biochem..

[143] Yan X.D., Pan L.Y., Yuan Y., Lang J.H., Mao N. (2007). Identification of platinum-resistance associated proteins through proteomic analysis of human ovarian cancer cells and their platinum-resistant sublines. J. Proteome Res..

[144] Zhang H.S., Zhao J.W., Wang H., Zhang H.Y., Ji Q.Y., Meng L.J., Xing F.J., Yang S.T., Wang Y. (2014). LIM kinase 1 is required for insulin-dependent cell growth of osteosarcoma cell lines. Mol. Med. Rep..

[145] Jiang N., Kham S.K., Koh G.S., Suang Lim J.Y., Ariffin H., Chew F.T., Yeoh A.E. (2011). Identification of prognostic protein biomarkers in childhood acute lymphoblastic leukemia (ALL). J. Proteom..

[146] Yan H., Yang K., Xiao H., Zou Y.J., Zhang W.B., Liu H.Y. (2012). Over-expression of cofilin-1 and phosphoglycerate kinase 1 in astrocytomas involved in pathogenesis of radioresistance. CNS Neurosci. Ther..

[147] Du H.Q., Chen L., Wang Y., Wang L.J., Yan H., Liu H.Y., Xiao H. (2015). Increasing radiosensitivity with the downregulation of cofilin-1 in U251 human glioma cells. Mol. Med. Rep..

[148] Patil K.S., Basak I., Pal R., Ho H.P., Alves G., Chang E.J., Larsen J.P., Møller S.G. (2015). A Proteomics Approach to Investigate miR-153-3p and miR-205-5p Targets in Neuroblastoma Cells. PLoS ONE.

[149] Lu L., Fu N.I., Luo X.U., Li X.Y., Li X.P. (2015). Overexpression of cofilin 1 in prostate cancer and the corresponding clinical implications. Oncol. Lett..

[150] Yang Z.L., Miao X., Xiong L., Zou Q., Yuan Y., Li J., Liang L., Chen M., Chen S. (2013). CFL1 and Arp3 are biomarkers for metastasis and poor prognosis of squamous cell/adenosquamous carcinomas and adenocarcinomas of gallbladder. Cancer Investig..

[151] Nagai S., Moreno O., Smith C.A., Ivanchuk S., Romagnuolo R., Golbourn B., Weeks A., Seol H.J., Rutka J.T. (2011). Role of the cofilin activity cycle in astrocytoma migration and invasion. Genes Cancer.

[152] Tsai C.H., Lin L.T., Wang C.Y., Chiu Y.W., Chou Y.T., Chiu S.J., Wang H.E., Liu R.S., Wu C.Y., Chan P.C. (2015). Over-expression of cofilin-1 suppressed growth and invasion of cancer cells is associated with up-regulation of let-7 microRNA. Biochim. Biophys. Acta.

[153] Chung H., Kim B., Jung S.H., Won K.J., Jiang X., Lee C.K., Lim S.D., Yang S.K., Song K.H., Kim H.S. (2013). Does phosphorylation of cofilin affect the progression of human bladder cancer?. BMC Cancer.

[154] Peng X.C., Gong F.M., Zhao Y.W., Zhou L.X., Xie Y.W., Liao H.L., Lin H.J., Li Z.Y., Tang M.H., Tong A.P. (2011). Comparative proteomic approach identifies PKM2 and cofilin-1 as potential diagnostic, prognostic and therapeutic targets for pulmonary adenocarcinoma. PLoS ONE.

[155] Guan M., Chen X., Ma Y., Tang L., Guan L., Ren X., Yu B., Zhang W., Su B. (2015). MDA-9 and GRP78 as potential diagnostic biomarkers for early detection of melanoma metastasis. Tumour Biol..

[156] Zheng Y., Fang Y., Li S., Zheng B. (2013). Detection of plasma cofilin protein for diagnosis of lung cancer. Nan Fang Yi Ke Da Xue Xue Bao.

[157] Xiao P., Ma T., Zhou C., Xu Y., Liu Y., Zhang H. (2016). Anticancer effect of docetaxel induces apoptosis of prostate cancer via the cofilin-1 and paxillin signaling pathway. Mol. Med. Rep..

[158] Becker M., de Bastiani M.A., Muller C.B., Markoski M.M., Castro M.A., Klamt F. (2014). High cofilin-1 levels correlate with cisplatin resistance in lung adenocarcinomas. Tumour Biol..

[159] Wang W., Mouneimne G., Sidani M., Wyckoff J., Chen X., Makris A., Goswami S., Bresnick A.R., Condeelis J.S. (2006). The activity status of cofilin is directly related to invasion, intravasation, and metastasis of mammary tumors. J. Cell Biol..

[160] Ito T., Taniguchi H., Fukagai K., Okamuro S., Kobayashi A. (2015). Inhibitory mechanism of FAT4 gene expression in response to actin dynamics during Src-induced carcinogenesis. PLoS ONE.

[161] Dubois F., Keller M., Calvayrac O., Soncin F., Hoa L., Hergovich A., Parrini M.C., Mazieres J., Vaisse-Lesteven M., Camonis J. (2016). RASSF1A Suppresses the Invasion and Metastatic Potential of Human Non-Small Cell Lung Cancer Cells by Inhibiting YAP Activation through the GEF-H1/RhoB Pathway. Cancer Res..

[162] Migocka-Patrzałek M., Makowiecka A., Nowak D., Mazur A.J., Hofmann W.A., Malicka-Błaszkiewicz M. (2015). β- and γ-Actins in the nucleus of human melanoma A375 cells. Histochem. Cell Biol..

[163] Wang Y., Kuramitsu Y., Kitagawa T., Baron B., Yoshino S., Maehara S., Maehara Y., Oka M., Nakamura K. (2015). Cofilin-phosphatase slingshot-1L (SSH1L) is over-expressed in pancreatic cancer (PC) and contributes to tumor cell migration. Cancer Lett..

[164] Kurita S., Watanabe Y., Gunji E., Ohashi K., Mizuno K. (2008). Molecular dissection of the mechanisms of substrate recognition and F-actin-mediated activation of cofilin-phosphatase Slingshot-1. J. Biol. Chem..

[165] Peterburs P., Heering J., Link G., Pfizenmaier K., Olayioye M.A., Hausser A. (2009). Protein kinase D regulates cell migration by direct phosphorylation of the cofilin phosphatase slingshot 1 like. Cancer Res..

[166] McConnell B.V., Koto K., Gutierrez-Hartmann A. (2011). Nuclear and cytoplasmic LIMK1 enhances human breast cancer progression. Mol. Cancer.

[167] Yoshioka K., Foletta V., Bernard O., Itoh K. (2003). A role for LIM kinase in cancer invasion. Proc. Natl. Acad. Sci. USA.

[168] Bagheri-Yarmand R., Mazumdar A., Sahin A.A., Kumar R. (2006). LIM kinase 1 increases tumor metastasis of human breast cancer cells via regulation of the urokinase-type plasminogen activator system. Int. J. Cancer.

[169] Pawlak G., Helfman D.M. (2002). MEK mediates v-Src-induced disruption of the actin cytoskeleton via inactivation of the Rho-ROCK-LIM kinase pathway. J. Biol. Chem..

[170] Vitolo M.I., Boggs A.E., Whipple R.A., Yoon J.R., Thompson K., Matrone M.A., Cho E.H., Balzer E.M., Martin S.S. (2013). Loss of PTEN induces microtentacles through PI3K-independent activation of cofilin. Oncogene.

[171] Beck A.H., Lee C.H., Witten D.M., Gleason B.C., Edris B., Espinosa I., Zhu S., Li R., Montgomery K.D., Marinelli R.J. (2010). Discovery of molecular subtypes in leiomyosarcoma through integrative molecular profiling. Oncogene.

[172] Demicco E.G., Boland G.M., Brewer Savannah K.J., Lusby K., Young E.D., Ingram D., Watson K.L., Bailey M., Guo X., Hornick J.L. (2015). Progressive loss of myogenic differentiation in leiomyosarcoma has prognostic value. Histopathology.

[173] Wang Y., Kuramitsu Y., Ueno T., Suzuki N., Yoshino S., Iizuka N., Zhang X., Oka M., Nakamura K. (2011). Differential expression of up-regulated cofilin-1 and down-regulated cofilin-2 characteristic of pancreatic cancer tissues. Oncol. Rep..

[174] Erkutlu I., Cigiloglu A., Kalender M.E., Alptekin M., Demiryurek A.T., Suner A., Ozkaya E., Ulasli M., Camci C. (2013). Correlation between Rho-kinase pathway gene expressions and development and progression of glioblastoma multiforme. Tumour Biol..

[175] Luo D., Wilson J.M., Harvel N., Liu J., Pei L., Huang S., Hawthorn L., Shi H. (2013). A systematic evaluation of miRNA:mRNA interactions involved in the migration and invasion of breast cancer cells. J. Transl. Med..

[176] Schwickert A., Weghake E., Bruggemann K., Engbers A., Brinkmann B.F., Kemper B., Seggewi J., Stock C., Ebnet K., Kiesel L. (2015). MicroRNA miR-142-3p Inhibits Breast Cancer Cell Invasiveness by Synchronous Targeting of WASL, Integrin Alpha V, and Additional Cytoskeletal Elements. PLoS ONE.

[177] Klose T., Abiatari I., Samkharadze T., de Oliveira T., Jäger C., Kiladze M., Valkovskaya N., Friess H., Michalski C.W., Kleeff J. (2012). The actin binding protein destrin is associated with growth and perineural invasion of pancreatic cancer. Pancreatology.

[178] Meacham C.E., Ho E.E., Dubrovsky E., Gertler F.B., Hemann M.T. (2009). In vivo RNAi screening identifies regulators of actin dynamics as key determinants of lymphoma progression. Nat. Genet..

[179] Baniwal S.K., Khalid O., Gabet Y., Shah R.R., Purcell D.J., Mav D., Kohn-Gabet A.E., Shi Y., Coetzee G.A., Frenkel B. (2010). Runx2 transcriptome of prostate cancer cells: Insights into invasiveness and bone metastasis. Mol. Cancer.

[180] Samaeekia R., Adorno-Cruz V., Bockhorn J., Chang Y.F., Huang S., Prat A., Ha N., Kibria G., Huo D., Zheng H. (2016). MicroRNA-206 inhibits stemness and metastasis of breast cancer by targeting MKL1/IL11 pathway. Clin. Cancer Res..

[181] Ishikawa R., Hayashi K., Shirao T., Xue Y., Takagi T., Sasaki Y., Kohama K. (1994). Drebrin, a development-associated brain protein from rat embryo, causes the dissociation of tropomyosin from actin filaments. J. Biol. Chem..

[182] Asada H., Uyemura K., Shirao T. (1994). Actin-binding protein, drebrin, accumulates in submembranous regions in parallel with neuronal differentiation. J. Neurosci. Res..

[183] Terakawa Y., Agnihotri S., Golbourn B., Nadi M., Sabha N., Smith C.A., Croul S.E., Rutka J.T. (2013). The role of drebrin in glioma migration and invasion. Exp. Cell Res..

[184] Xu S.Q., Buraschi S., Morcavallo A., Genua M., Shirao T., Peiper S.C., Gomella L.G., Birbe R., Belfiore A., Iozzo R.V. (2015). A novel role for drebrin in regulating progranulin bioactivity in bladder cancer. Oncotarget.

[185] Mitra R., Lee J., Jo J., Milani M., McClintick J.N., Edenberg H.J., Kesler K.A., Rieger K.M., Badve S., Cummings O.W. (2011). Prediction of postoperative recurrence-free survival in non-small cell lung cancer by using an internationally validated gene expression model. Clin. Cancer Res..

[186] Lin Q., Tan H.T., Lim T.K., Khoo A., Lim K.H., Chung M.C. (2014). iTRAQ analysis of colorectal cancer cell lines suggests Drebrin (DBN1) is overexpressed during liver metastasis. Proteomics.

[187] Yang C., Li Z., Shi Z., He K., Tian A., Wu J., Zhang Y., Li Z. (2014). Regulation of cell survival by the HIP-55 signaling network. Mol. Biosyst..

[188] Boateng L.R., Bennin D., de Oliveira S., Huttenlocher A. (2016). Mammalian Actin-binding Protein-1/Hip-55 Interacts with FHL2 and Negatively Regulates Cell Invasion. J. Biol. Chem..

[189] Higashida H., Kato T., Kano-Tanaka K., Okuya M., Miyake A., Tanaka T. (1981). Proliferation and synapse formation of neuroblastoma glioma hybrid cells: Effects of glia maturation factor. Brain Res..

[190] Lim R., Zaheer A., Yorek M.A., Darby C.J., Oberley L.W. (2000). Activation of nuclear factor-kappaB in C6 rat glioma cells after transfection with glia maturation factor. J. Neurochem..

[191] Kuang X.Y., Jiang X.F., Chen C., Su X.R., Shi Y., Wu J.R., Zhang P., Zhang X.L., Cui Y.H., Ping Y.F. (2015). Expressions of glia maturation factor-β by tumor cells and endothelia correlate with neovascularization and poor prognosis in human glioma. Oncotarget.

[192] Zaheer A., Knight S., Zaheer A., Ahrens M., Sahu S.K., Yang B. (2008). Glia maturation factor overexpression in neuroblastoma cells activates glycogen synthase kinase-3beta and caspase-3. Brain Res..

[193] Wada T., Yamashita Y., Saga Y., Takahashi K., Koinuma K., Choi Y.L., Kaneda R., Fujiwara S., Soda M., Watanabe H. (2009). Screening for genetic abnormalities involved in ovarian carcinogenesis using retroviral expression libraries. Int. J. Oncol..

[194] Li Y.L., Ye F., Cheng X.D., Hu Y., Zhou C.Y., Lü W.G., Xie X. (2010). Identification of glia maturation factor beta as an independent prognostic predictor for serous ovarian cancer. Eur. J. Cancer.

[195] Zuo P., Ma Y., Huang Y., Ye F., Wang P., Wang X., Zhou C., Lu W., Kong B., Xie X. (2014). High GMFG expression correlates with poor prognosis and promotes cell migration and invasion in epithelial ovarian cancer. Gynecol. Oncol..

[196] Nakatsura T., Senju S., Ito M., Nishimura Y., Itoh K. (2002). Cellular and humoral immune responses to a human pancreatic cancer antigen, coactosin-like protein, originally defined by the SEREX method. Eur. J. Immunol..

[197] Cecconi D., Astner H., Donadelli M., Palmieri M., Missiaglia E., Hamdan M., Scarpa A., Righetti P.G. (2003). Proteomic analysis of pancreatic ductal carcinoma cells treated with 5-aza-2′-deoxycytidine. Electrophoresis.

[198] Oh J.E., Karlmark Raja K., Shin J.H., Pollak A., Hengstschläger M., Lubec G. (2006). Cytoskeleton changes following differentiation of N1E-115 neuroblastoma cell line. Amino Acids.

[199] Jeong H.C., Kim G.I., Cho S.H., Lee K.H., Ko J.J., Yang J.H., Chung K.H. (2011). Proteomic analysis of human small cell lung cancer tissues: Up-regulation of coactosin-like protein-1. J. Proteome Res..

